# Modulated self-assembly of metal–organic frameworks†

**DOI:** 10.1039/d0sc01356k

**Published:** 2020-04-06

**Authors:** Ross S. Forgan

**Affiliations:** WestCHEM School of Chemistry, University of Glasgow Glasgow UK ross.forgan@glasgow.ac.uk

## Abstract

Exercising fine control over the synthesis of metal–organic frameworks (MOFs) is key to ensuring reproducibility of physical properties such as crystallinity, particle size, morphology, porosity, defectivity, and surface chemistry. The principle of modulated self-assembly – incorporation of modulator molecules into synthetic mixtures – has emerged as the primary means to this end. This perspective article will detail the development of modulated synthesis, focusing primarily on coordination modulation, from a technique initially intended to cap the growth of MOF crystals to one that is now used regularly to enhance crystallinity, control particle size, induce defectivity and select specific phases. The various mechanistic driving forces will be discussed, as well as the influence of modulation on physical properties and how this can facilitate potential applications. Modulation is also increasingly being used to exert kinetic control over self-assembly; examples of phase selection and the development of new protocols to induce this will be provided. Finally, the application of modulated self-assembly to alternative materials will be discussed, and future perspectives on the area given.

## Abstract 2

This Perspective gives an overview of the modulated self-assembly of MOFs – incorporating additives and alternative precursors into syntheses – focusing on its varying influences on crystallization mechanisms, physical properties, and applications.

## Graphical abstract

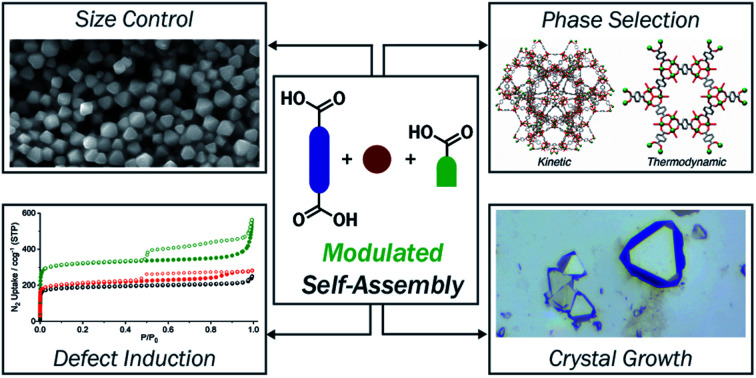

## 1. Introduction

Metal–organic frameworks (MOFs) – coordination polymers wherein metal ions or clusters are connected by organic struts into extended network solids with potential porosity – are currently amongst the most researched materials in chemistry and materials science,^1^ with ∼70 000 structures reported in the Cambridge Structural Database by 2017.^2^ As this very large number of published structures suggests, MOFs can be relatively easy to prepare. Conventional solvothermal syntheses involve the combination of a metal source and an organic linker (or linkers), usually in a formamide solvent, which is heated to induce release of a base by thermal decomposition, thus initiating self-assembly, although a number of alternative protocols have been successfully developed.^3^ Whilst this methodology is often successful in delivering crystals that allow characterization of materials by single crystal X-ray diffraction, there are a number of physical properties, such as phase purity, interpenetration, porosity, defectivity, particle size, morphology and surface chemistry, that must be finely controlled to ensure that MOFs are optimized towards particular applications such as gas storage,^4^ catalysis,^5^ sensing^6^ and drug delivery.^7^ Additionally, the chemical stability afforded to MOFs linked by high valent metals through the kinetic inertness of their metal–ligand bonding can also hinder the synthesis of highly crystalline material.^8^ As a consequence, modulated synthesis has emerged as a protocol to exert control over the self-assembly of MOFs and produce materials with desired properties.

**Fig. 1:**
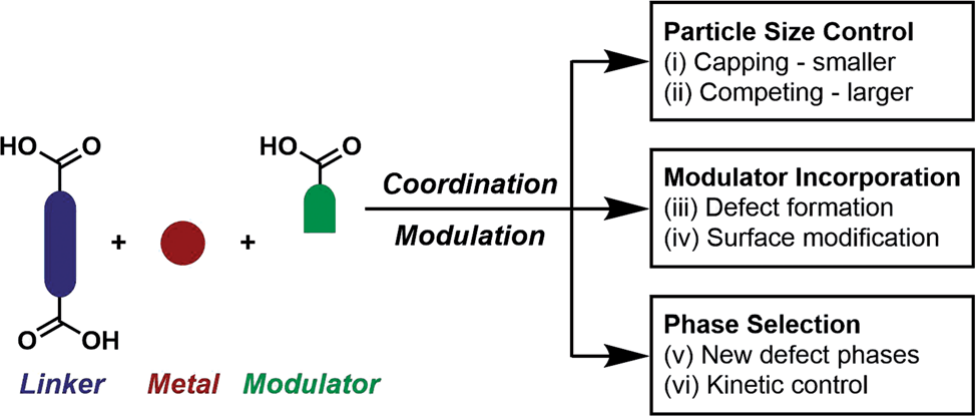
Schematic of coordination modulation, with a range of different potential outcomes. Note that these can occur concurrently, for example (i) aligns with (iv), (iii) can align with (v), and (ii) can align with (vi).

**Fig. 2:**
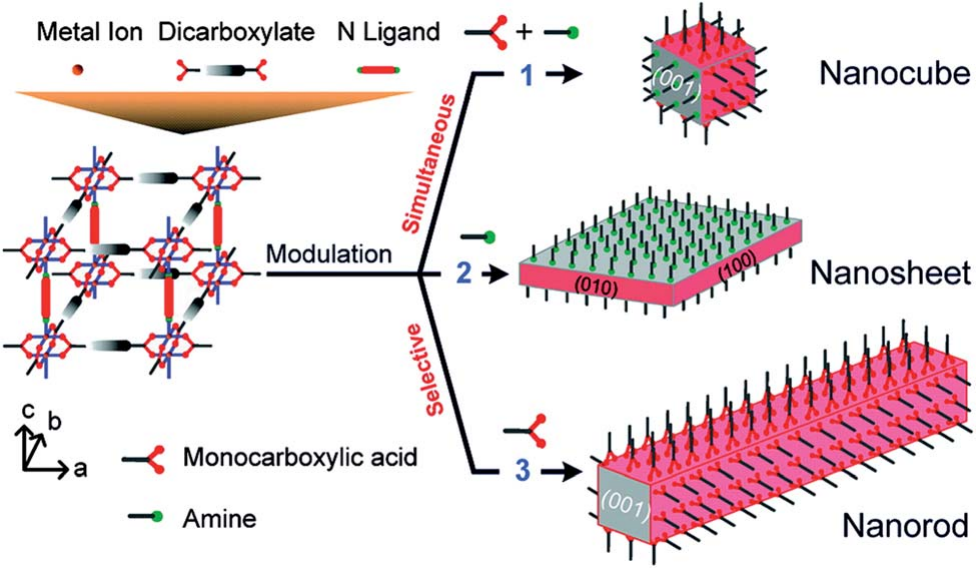
Schematic of face-selective coordination modulation of [Cu_2_(1,4-ndc)_2_(dabco)]_*n*_. Reprinted with permission from ref. 12. Copyright (2012) American Chemical Society.

The incorporation of monotopic ligands that mimic the functionality of the multitopic MOF linkers into solvothermal syntheses (specifically named coordination modulation) can have a range of effects, but it was first conceived as a method to control particle size (effect (i) in Fig. 1). In 2007, Fischer *et al.* incorporated *p*-perfluoromethylbenzoic acid into solvothermal syntheses of MOF-5, [Zn_4_O(1,4-bdc)_3_]_*n*_ where 1,4-bdc is 1,4-benzenedicarboxylate (chemical structures of all modulators and linkers are provided in Tables S1 and S2 in the ESI†), to examine the self-assembly of the material by time-resolved static light scattering (SLS). Incorporation of the modulator reduced crystallite size from 350 nm to around 100–150 nm, depending on the ratio of modulator to MOF precursors, and yielded stable colloidal solutions. It was postulated that the modulator competed with the 1,4-bdc ligands for the Zn^2+^ cations and that this capping approach would become a general protocol for controlling particle size during synthesis.^9^ The coordinative capping hypothesis was further evidenced by a subsequent report from Kitagawa *et al.*, where the term “coordination modulation” was first introduced,^10^ examining the modulated self-assembly of the pillar-layer MOF [Cu_2_(1,4-ndc)_2_(dabco)]_*n*_, where 1,4-ndc = 1,4-naphthalenedicarboxylate and dabco = 1,4-diazabicyclo[2.2.2]octane. This material has tetragonal symmetry and presents crystal facets terminated by either carboxylate units from the 1,4-ndc linkers or N-donor atoms from the dabco pillar, as a consequence of its [Cu_2_(R^1^CO_2_)_4_(R^2^N)_2_] paddlewheel secondary building units (SBUs). The differing functionalities allow anisotropic growth through addition of a modulator complementary to the chemistry at particular crystal faces. Addition of acetic acid to the syntheses results in nanorod formation with growth along the [001] axis, which is the dabco-presenting face; the acetic acid is effectively capping the carboxylate-presenting face.^11^ This methodology was subsequently built upon by modulating the synthesis of the same MOF with pyridine, where the N-donor modulator in this case capped the [001] direction and resulted in the formation of square nanosheets. Modulation with both pyridine and acetic acid effectively capped crystal growth in all directions and resulted in a decrease in particle size and formation of nanocubes (Fig. 2).^12^

The coordinative interaction of modulators with growing MOF crystals was also demonstrated in the particle size and morphological control over HKUST-1, [Cu_3_(1,3,5-btc)_2_]_*n*_, where 1,3,5-btc = 1,3,5-benzenetricarboxylate. Addition of increasing quantities of dodecanoic acid modulator to microwave syntheses carried out in *n*-butanol modified particle size from the nanometre to the micron scale,^13^ and could also tune crystal morphology from octahedron, to cuboctahedron, to truncated cube, and finally to cube. Computational modeling indicated that modulation inhibited growth in the [100] direction, while it was posited that the modulator was involved in the preformation of SBUs which enhanced growth kinetics in the [111] direction.^14^ Similar face-selective modulation has been observed in the acetic acid modulated synthesis of MIL-53(Al)–NH_2_; in this material, [Al(OH)(2-NH_2_-1,4-bdc)]_*n*_, the carboxylate functionality projects in two dimensions with one dimensional Al–OH chains in the third. Face-selective coordination modulation results in microneedles, with the major axis running along the [001] plane, coincident with the direction of the Al–OH chains.^15^

**Fig. 3:**
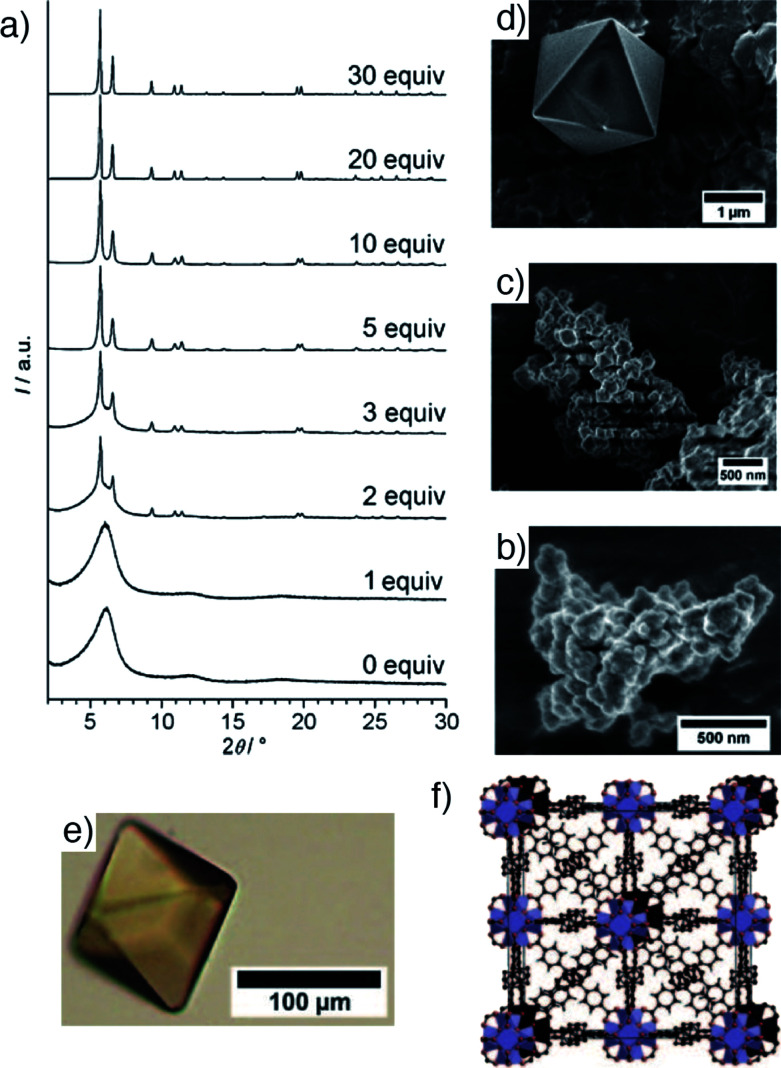
(a) Stacked powder X-ray diffractograms of UiO-67 samples showing enhancement of crystallinity with addition of benzoic acid modulator to syntheses. Accompanying SEM images of samples modulated by (b) 0 equivalents, (c) 3 equivalents, and (d) 30 equivalents of benzoic acid. (e) A single crystal of UiO-68-NH_2_, prepared through benzoic acid modulation. (f) Packing image from the crystal structure of UiO-68-NH_2_. Reprinted (adapted) with permission from ref. 16. Copyright (2011) John Wiley & Sons.

A major breakthrough in coordination modulation was its application by Behrens *et al.* to the self-assembly of Zr MOFs.^16^ Having first been reported in 2008,^17^ with structures solved from powder X-ray diffraction data, it was shown that modulation could dramatically enhance crystallinity and particle size of Zr MOFs of the UiO-66 isoreticular series, which have formula [Zr_6_O_4_(OH)_4_L_6_]_*n*_, where L is a linear dicarboxylate linker. Modulation with benzoic or acetic acid enhanced both particle size and porosity in UiO-66 (L = 1,4-bdc) and UiO-67 (L = biphenyl-4,4′-dicarboxylate, bpdc, Fig. 3a–d), and allowed access to 100 μm single crystals of UiO-68-NH_2_ (L = 2′-amino-1,1′:4,1′′-terphenyl-4,4′′-dicarboxylate, or tpdc-NH_2_, Fig. 3e) to confirm its structure by single crystal X-ray diffraction analysis (Fig. 3f). It was hypothesized that, in this case, coordination modulation offers kinetic control over self-assembly, slowing down crystallization to enhance the quality of the MOF through coordinative competition for the metal cations between the linkers and modulators.

With the ability of coordination modulation to not only tune the physical properties of previously known MOFs, but to enhance and promote the formation of new phases, firmly established, the technique subsequently become commonplace in the self-assembly of MOFs. This Perspective Article will highlight the versatility of modulation in the self-assembly of MOFs and related porous materials, covering its fundamental effects on physical properties and the applications of MOFs that fine control over these has enabled, while examining mechanistics and offering future perspectives.

## 2. Particle size control

One of the key outcomes of syntheses utilizing coordination modulation is particle size control, resulting in MOF crystallites ranging from the nanometre to the millimetre scale, and to rationalize these results it is necessary to consider the proposed mechanism(s) induced by modulation. The hypothesis behind the archetypal studies described above was that modulators would inhibit particle growth by coordinating to the inorganic SBUs at crystal faces, therefore inhibiting coordination polymerisation in a manner directly analogous to terminating the growth of covalent, organic polymer chains. However, the lability of the coordination bond, in contrast to irreversible covalent carbon–carbon bonds, means that modulators are also thought to compete with the ligands for the metal ions and thus introduce some reversibility and kinetic control over MOF formation. They can also modify reaction pH, by competing for the bases released in solvothermal synthesis and introducing additional protons to inhibit linker deprotonation. Brozek *et al.* have therefore proposed four main driving forces that control MOF particle size: (i) linker deprotonation, (ii) modulator deprotonation, (iii) linker complexation, and (iv) particle termination, or capping.^18^ Additionally, modulators may also pre-form the multinuclear SBUs that connect MOFs: utilizing pre-formed clusters is already a known approach for synthesis of a range of MOFs,^19–24^ which pre-dates coordination modulation itself,^19^ and there is evidence that these clusters remain intact during MOF self-assembly, rather than simply acting as an alternative metal source.^25^ As such, understanding of the various driving forces in MOF self-assembly is limited by the complexity of the pre-crystallisation solution speciation, and introduction of additional complexity in the shape of modulators makes analysis challenging. Nevertheless, a number of groups have attempted to monitor this, both by *ex situ* examination of the modulated products and *in situ* analysis of the self-assembly processes.^26,27^

### 2.1. Mechanistic investigations

The chemical nature of the modulator will clearly affect the physical properties of the modulated MOF. For example, in the carboxylic acid modulated syntheses of MIL-101(Cr), a large pore material which has formula [Cr_3_O(1,4-bdc)_3_(OH_2_)_2_X]_*n*_ (X = a monocounterion such as OH^−^, Cl^−^, or F^−^), particle size increases as the p*K*_a_ of the modulator decreases, from 19(4) nm with stearic acid (p*K*_a_ = 10.15) to 73(8) nm with perfluorobenzoic acid (p*K*_a_ = 1.60). This suggests that modulators which are more likely to be deprotonated under the reaction conditions (*i.e.* those with higher p*K*_a_) are more likely to interact with the growing crystal through coordination to the metal sites and cap growth.^28^ This hypothesis is also borne out in the use of sodium carboxylate salts as modulators rather than the corresponding free acids; reduced particle size was achieved for sodium acetate modulation of MIL-68(In), [In(OH)(1,4-bdc)]_*n*_,^29^ as well as sodium acetate and sodium formate modulation of HKUST-1.^30^ In the latter case, sodium formate yielded smaller particles than sodium acetate; when added as a salt rather than an acid, the more basic formate seemingly interacts more strongly with the growing crystals than acetate and more effectively caps crystal growth.

**Fig. 4:**
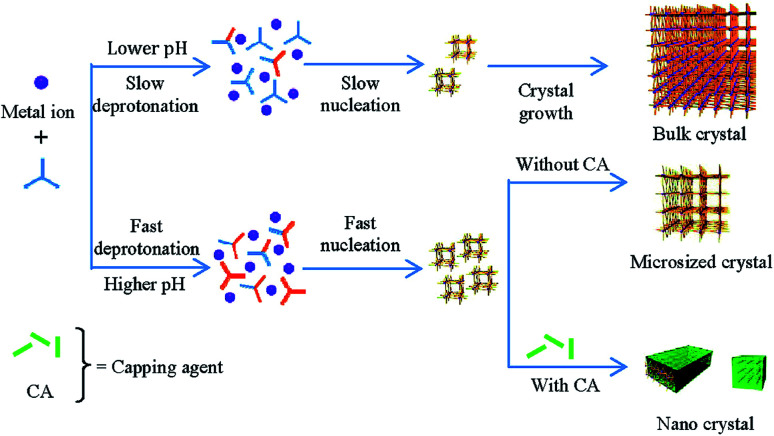
Proposed mechanisms from Zhang *et al.* to explain the dual roles of pH control and coordination modulation in MOF particle size control. Reprinted with permission from ref. 32. Copyright (2012) American Chemical Society.

The effect of modulator addition on synthesis pH should also be taken into account. In the sodium acetate modulated synthesis of [Dy(1,3,5-btc)(H_2_O)]_*n*_, particle size reduction, accompanied by changes in particle morphology, could be related to the amount of sodium acetate added.^31^ Using acetic acid as a modulator alongside trimethylamine to tune reaction pH to ∼6 generated similar particle size decreases, but tuning pH alone did not. It was proposed that linker deprotonation occurs more rapidly at higher pH, inducing faster nucleation, and that capping modulators can terminate crystal growth and produce monodisperse downsized particles; a synergistic effect between pH control and crystal capping appears to be the mechanism for the very efficient particle size reductions observed with carboxylate salts. At lower pH, deprotonation is slower and so nucleation occurs over a larger time period, with subsequent crystal growth producing larger particles and a larger distribution of sizes (Fig. 4).^32^

Brozek *et al.* have carried out a comprehensive analysis on the self-assembly of nanoscale MOFs, evaluating a number of factors that influence particle size, including the amount of modulator added to syntheses. They suggest that adding small amounts of acidic modulators serves to decrease particle size, but as the modulator ratio increases the additional proton content hinders linker deprotonation and introduces excessive coordination competition, increasing particle size.^18^ An important additional aspect to consider when attempting to control particle size is the p*K*_a_ of the modulator relative to that of the linker itself, as the conjugate basicity of the deprotonated carboxylate modulator will mediate the level of coordinative competition. It could be expected that modulators with p*K*_a_ values similar to those of the linkers will facilitate growth of large particles and single crystals, which may explain the widespread use of acetic acid (p*K*_a_ = 4.76) and benzoic acid (p*K*_a_ = 4.20) in the isolation of single crystals of MOFs linked by 1,4-bdc (first p*K*_a_ = 4.82), prime examples being UiO-66 and its isoreticular analogues modulated with benzoic acid.^16,33^ Clearly, a number of parameters must be considered when selecting a modulator, and they often result in competing influences, meaning fine tuning of syntheses are often required.

**Fig. 5:**
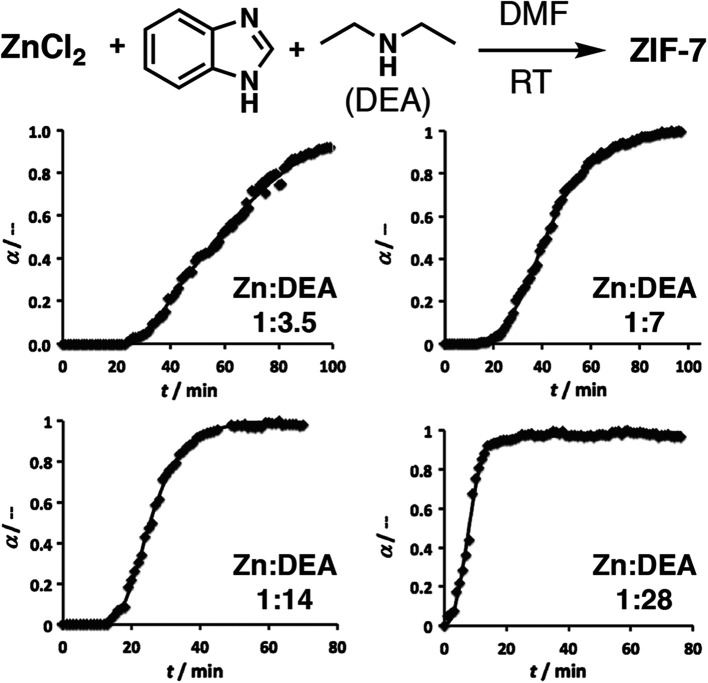
Experimental (symbols) and calculated (line) profiles of the evolution of the Bragg peak at *Q* = 5.1 nm^−1^ from *in situ* wide-angle X-ray scattering data during the diethylamine (DEA) modulated self-assembly of ZIF-7, showing an increase in crystallization rate with addition of more base. Reprinted (adapted) with permission from ref. 34. Copyright (2013) John Wiley & Sons.

An examination of the pH effect in the crystallization of ZIF-7, [Zn(bIm)_2_]_*n*_, where bIm = benzimidazole, was carried out by *in situ* X-ray scattering experiments, using diethylamine as a pH modulator. It should be noted that the term modulator is used broadly in the literature for agents added into syntheses to “modulate” crystallization and self-assembly. Those that do not coordinate, and only modify pH, are therefore often referred to as pH or proton modulators. For this synthesis, both wide and small angle X-ray scattering experiments were used to monitor crystallization, for which both the onset of nucleation and crystal growth occurred more rapidly as more diethylamine was added (Fig. 5). After longer reaction periods, these smaller crystals seemed better able to coalesce into larger structures, as larger particles were found in the syntheses containing more diethylamine, suggesting an alternative mechanism for growth compared to the metal-carboxylate MOFs discussed previously. Extended X-ray absorption spectroscopy confirmed the diethylamine did not coordinate to the zinc and acted solely as a pH modulator.^34^

The crystallization of nanoparticles of ZIF-8, [Zn(MeIm)_2_]_*n*_, where MeIm = 2-methylimidazole, with modulation by 1-methylimidazole, sodium formate, and *n*-butylamine at room temperature in methanol, was studied by *in situ* light scattering techniques. Both modulated and unmodulated syntheses were found to be characterized by relatively slow nucleation and fast crystal growth. The highly basic *n*-butylamine as modulator yielded much smaller particles (9–55 nm depending on conditions) than unmodulated syntheses (∼63 nm), but 1-methylimidazole and sodium formate gave monodisperse, micron scale, well-defined rhombic dodecahedra. Static light scattering showed that both modulators resulted in lower nucleation rates and lower growth rates – 1-methylimidazole has a more significant effect as it is a better ligand than formate – clearly demonstrating the coordinative competition between modulator and linker.^35^ A further study of sodium formate modulation of ZIF-8 by *in situ* energy-dispersive X-ray diffraction (EDXRD), this time at high temperature, indicated that, under these conditions, formate acted as a pH modulator and not a coordinating modulator; crystallization is therefore rapid, but large, uncapped particles can be formed.^36^ The role of reaction temperature highlights the importance of controlling kinetic effects during modulation to mediate different driving forces that control self-assembly.

Modulated self-assembly of MOF-5 at room temperature, by combining pre-formed [Zn_4_O(C_6_H_5_COO)_6_] clusters with 1,4-bdc in DMF at room temperature alongside 4-decylbenzoic acid as modulator, was monitored by *in situ* small angle neutron scattering (SANS) to try to understand the mechanism of modulation. It was found that modulation slowed down both the initial formation of MOF particles and their growth rate, but had no effect on final particle size compared to unmodulated syntheses.^37^ This suggests inhibition either through competitive metal coordination or pH control, but a follow-up study from the same group where the 4-decylbenzoic acid modulator was added in a larger excess 5 minutes after the initial mixing of the reagents allowed identification of the modulator at crystallite surfaces by SANS. This was feasible by determining an appropriate mixture of deuterated and protonated DMF solvent which matched the neutron scattering contrast of MOF-5, and therefore allowing scattering by the large alkyl chains of the modulator to be identified.^38^ This provides direct evidence of the coordination of modulators to the outer surfaces of growing MOF particles and their control over crystal growth.

**Fig. 6:**
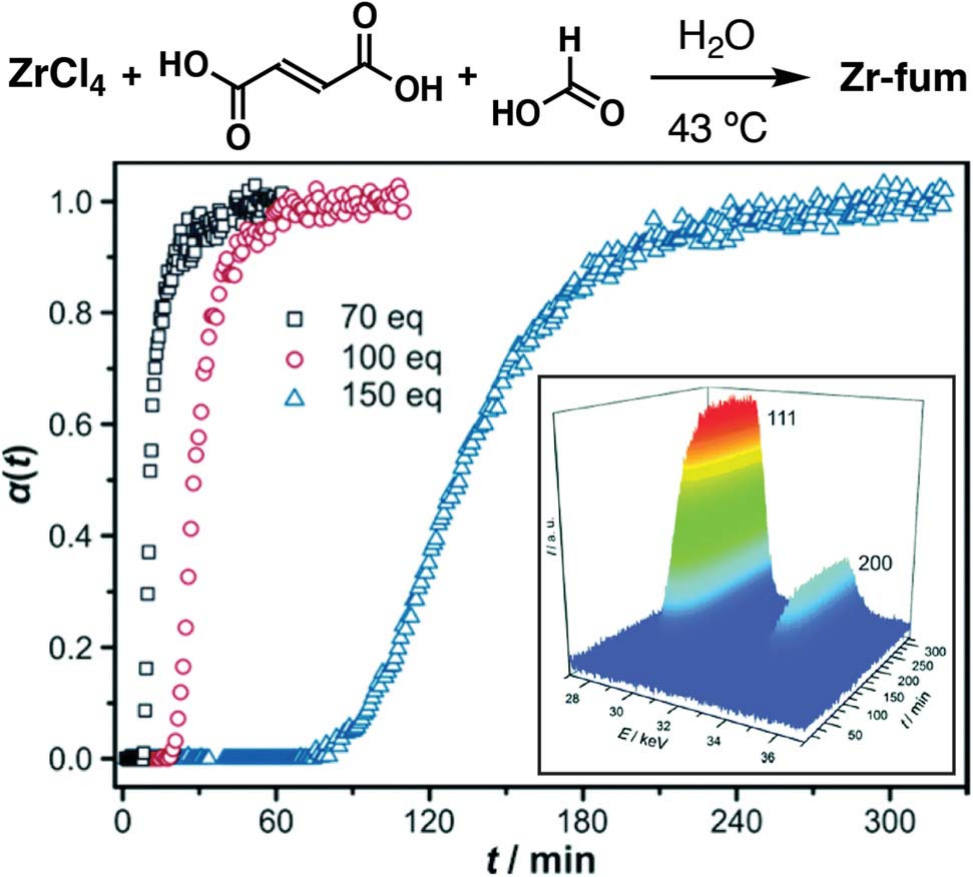
Time evolution of the crystallinity, α, of Zr-fum in aqueous, formic acid modulated syntheses at 43 °C, as assessed by *in situ* energy-dispersive X-ray diffraction analysis. Inset is a typical 3D plot of diffraction data used to construct α *vs.* t individual curves. Reprinted (adapted) with permission from ref. 39. Published (2014) by The Royal Society of Chemistry.

Also using *in situ* time-resolved EDXRD, Behrens *et al.* demonstrated that the aqueous synthesis of Zr-fum, the isoreticular analogue of UiO-66 containing fumarate as the linker (also known as MOF-801), could be significantly slowed by introduction of formic acid as modulator (Fig. 6a). Both nucleation and crystal growth rates were decreased, presumably due to formic acid competing both for proton and zirconium in the syntheses. In contrast, the DMF-based synthesis saw the opposite effect, with reaction rates increasing on formic acid addition, but induction of nucleation remaining constant (Fig. 6b). This was postulated to be a consequence of concomitant addition of incipient water with the formic acid, as water is required as the source of the bridging O^2−^ and OH^−^ cluster ligands; subsequent deliberate addition of water was found to rapidly increase nucleation rates, suggesting a more profound effect of water addition compared to coordination modulation.^39^

The consumption of formic acid modulator and DMF during an analogous synthesis of Zr-fum was monitored by *in situ* IR spectroscopy, which suggested significant incorporation of formate at defect sites in the resulting MOF (see Section 3) and also points towards coordinative interaction of the formate with the growing MOF.^40^ The role of water in the formation of UiO-66 has also been probed by EDXRD, specifically while investigating the use of HCl as a pH modulator. HCl was found to speed up the rate of reaction, contrary to the expected pH modulation effect whereby addition of a Brønsted acid would hinder linker deprotonation, but this was also found to be due to concomitant addition of water from the concentrated (37%) HCl used. An even greater effect was found using pure water, again suggesting that the effect of water addition on kinetics is more significant than pH modulation.^41^ We have recently used simple turbidity measurements to monitor nucleation of UiO-66, and our observations correlate these EDXRD studies. Modulation with benzoic acid, however, showed more complex behaviour; addition of 5 equivalents speeds up the onset of nucleation, in contrast to conventional modulation theory, while 10 or more equivalents slow it. This suggests a complex mixture of mechanisms underpin modulation rather than simple coordinative competition.^42^ The importance of water addition in these syntheses is underlined by an *in situ* pair distribution function analysis of cluster formation, which showed that the [Zr_6_O_4_(OH)_4_(RCO_2_)_12_] SBU that underpins most Zr MOFs can form in solution prior to ligand incorporation.^43^ Any additional modulator or ligand source that enhances this pre-clustering will likely increase reaction rates and modify particle sizes.

These mechanistic studies confirm that modulation can induce different mechanisms during self-assembly, particularly through coordination (competition, SBU preformation) and pH control (linker and modulator deprotonation), and these often compete against each other to control physical properties and reaction kinetics. Both metal coordination and pH control will be dictated by modulator p*K*_a_, as well as its relation to the linker p*K*_a_, and so this must be carefully considered when choosing an appropriate modulator. Temperature seems to amplify different driving forces with respect to each other, highlighting the importance of kinetics; for modulator or linker coordination, this will vary dramatically for different metals, and is another aspect to consider. Solvent choice is also important, both to control water content and release of potential modulators through thermal decomposition, *e.g.*, formate can be released by DMF reacting with water. Further systematic studies of modulation are required to clarify this complex mixture of pre-crystallisation processes.

### 2.2 Applications of modulated MOFs through size control

**Fig. 7:**
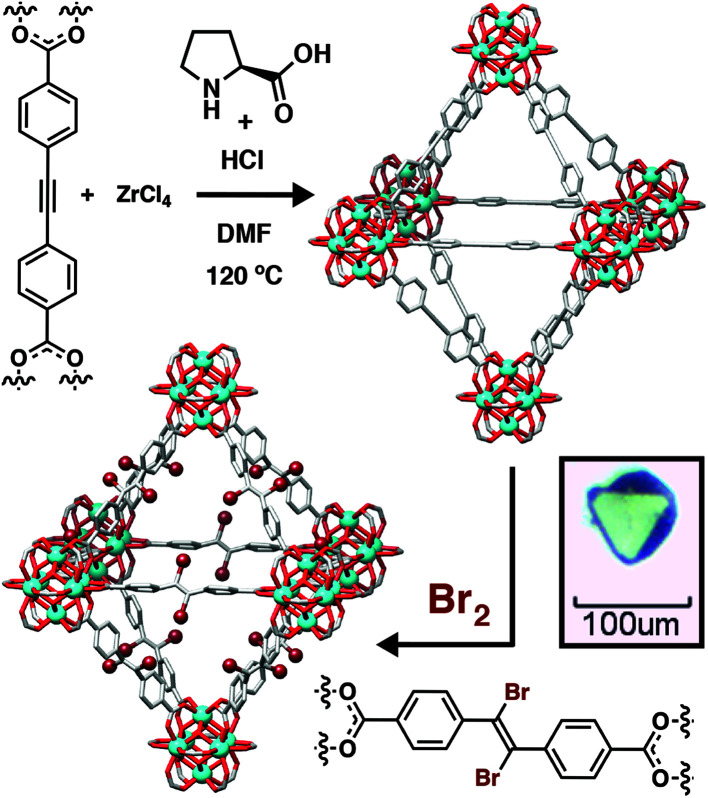
Schematic of l-proline modulated synthesis of a Zr MOF containing the edb linker as single crystals, and its subsequent single-crystal to single-crystal postsynthetic bromination. Reprinted (adapted) with permission from ref. 47. Further permissions related to the material should be directed to the American Chemical Society.

The ability to tune the particle size of MOF crystals using coordination modulation has allowed the study of physical properties by specialist techniques, and also uncovered unusual behavior and materials. Our group has pioneered the use of amino acids as modulators for Zr MOFs,^44,45^ with l-proline in particular proving highly effective at generating high-quality, defect-free single crystals of a range of systems. We have exploited unique access to these materials to probe mechanical compliance using high-pressure X-ray diffraction and nanoindentation on single crystals of UiO-67 and UiO-abdc, members of the UiO-66 isoreticular series containing bpdc and azobenzene-4,4′-dicarboxylate (abdc), respectively, as the linkers. These complementary techniques showed that UiO-abdc exhibited considerable mechanical compliance through an unusual bowing of the ligand, allowing up to 10% decrease in unit cell volume without compromising crystallinity, while UiO-67 was much more rigid.^46^ Access to high quality single crystals of mechanically and chemically robust MOFs has also allowed a detailed examination of single-crystal to single-crystal postsynthetic modification in those containing linkers with unsaturated C–C bonds as spacers (Fig. 7).^47,48^ Halogenation of these units occurs quantitatively, and the access to single crystals exclusively through coordination modulation has allowed unequivocal determination of the stereoselectivity by X-ray crystallography, showing that the topological restrictions on the ligands constrained within network solids result in different stereoselectivities compared to solution reactions. For example, bromination of the 4,4′-ethynylenedibenzoate (edb) linker in its corresponding Zr MOF of the UiO topology generated a purely *trans*-dibromoalkene product, but a mixture of *cis* and *trans* isomers in solution.^47^

As well as crystal growth, using coordination modulation for the downsizing of particles into the nanoscale brings the possibilities of new physical properties and applications,^49^ but it is important to note that modulation is one of many methodologies capable of controlling particle size in MOF self-assembly.^18^ Fine control of particle size can allow further assembly of MOF particles into functional superstructures. Modulation of a range of Zr MOFs, in particular UiO-66, with acetic acid in the presence of water yields nanoparticles in the 10–20 nm range, which formed organogels in the DMF reaction solvents. These could be converted to (i) xerogels, through air drying, (ii) monoliths, by drying ethanol-exchanged samples in air at elevated temperatures, or (iii) aerogels, by activation with supercritical CO_2_. Depending on superstructure, hierarchical porosity could be observed as a consequence of the voids between particles.^50^ Subsequent work extended this concept, using different drying temperatures and solvents to form UiO-66 monoliths with varying levels of mesoporosity from interparticle spacing. The acetic acid modulated synthesis ensures a relatively narrow particle size distribution, which is essential for densification into mechanically stable monoliths, and the ability to tune the mesoporosity resulted in materials with exceptional volumetric working capacities for storage and delivery of both CH_4_ and CO_2_.^51^

**Fig. 8:**
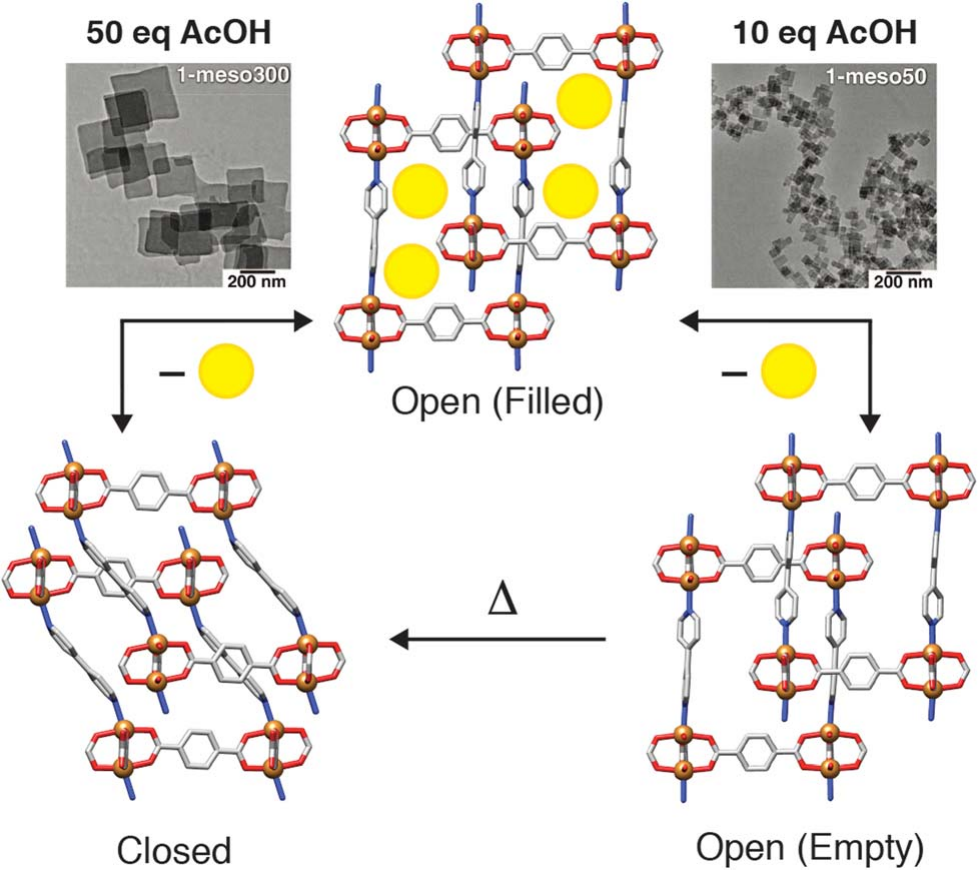
Schematic of size-induced shape memory in [Cu_2_(1,4-bdc)_2_(4,4′-bipy)]_*n*_ which occurs for 50 nm particles (right) but not >300 nm particles (left). Reprinted in part (adapted) with permission from ref. 53. Copyright (2013) The American Association for the Advancement of Science.

The effects of MOF particle size on adsorption phenomena, in particular breathing effects, are becoming more apparent through both experiment and simulation.^52^ One striking example is the induction of “shape memory” in the flexible framework [Cu_2_(1,4-bdc)_2_(4,4′-bipy)]_*n*_ (4,4′-bipy = 4,4′-bipyridine) through modulator assisted particle downsizing. Using acetic acid to control particle size, micrometre-sized crystals undergo a reversible phase transition from an open, DMF-solvated form, to a closed-pore structure on removal of the guest from the pores; resolvation with DMF regenerates the open-pore material. In contrast, nanoscale materials (∼50 nm in diameter) retained the open-pore form upon desolvation, presumably kinetically trapped in this metastable state, and could subsequently be converted to the closed pore form by thermal treatment (Fig. 8). These structural changes were also apparent in methanol adsorption isotherms of the nanoscale material, with gate-opening phenomena and hysteresis observed for the thermally treated, closed-pore MOF, but not for the metastable evacuated, but open-pore form.^53^

Coordination modulation also offers the possibility of simultaneously controlling particle size and surface chemistry, leading to obvious applications in drug delivery. These will be discussed in the context of surface modification through modulator incorporation in the coming section.

## 3. Modulator incorporation

One of the potential consequences of coordination modulation is significant incorporation of the modulator into the structure, as modulators have coordinating units complementary to the MOF linkers and therefore can replace them at secondary building units as charge-compensating defects. Understanding the defect chemistry of MOFs, in particular their deliberate induction through modulated self-assembly and the resultant effects on physical properties, has become an active research area in its own right.^54–59^ It is also possible to limit modulator incorporation to the outer surfaces of MOF particles, where the modulators effectively act as capping agents. These two possibilities will be discussed in the coming sections.

### 3.1 Modulation induced defectivity

**Fig. 9:**
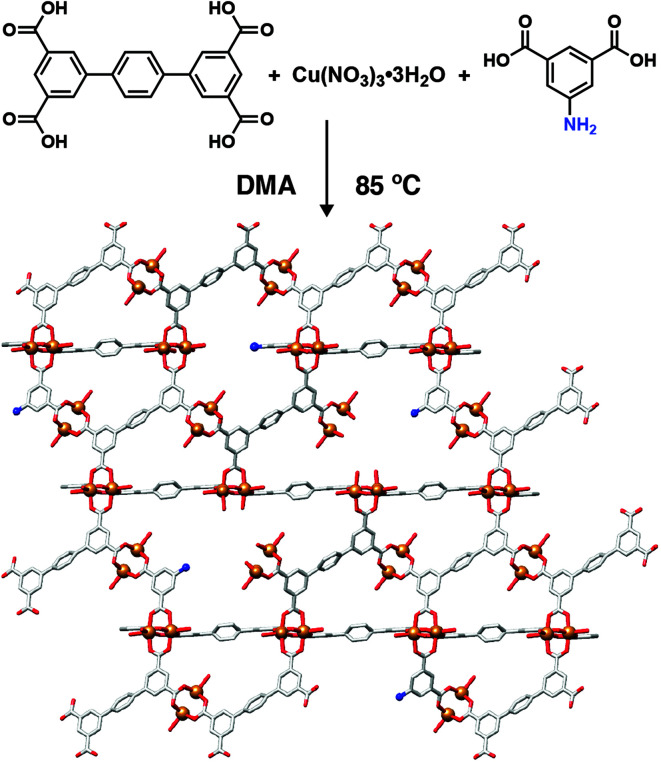
Schematic of 5-aminoisophthalic acid incorporation into NOTT-101 during coordination modulation to modify both pore texture and chemistry through defect formation.

Modulator incorporation throughout the MOF structure can occur (i) in an ordered manner, forming new, crystallographically ordered phases through coordination of modulators at each SBU in a specific fashion, (ii) in a completely disordered manner to distribute defects randomly throughout the structure, or (iii) somewhere inbetween, forming nanodomains within individual particles that have a different structure to the bulk material. Defects are more commonly found in MOFs with high-connectivity SBUs, for example the ubiquitous 12-connected Zr_6_ SBU, as it is thought that increased connectivity facilitates stability when modulators replace bridging ligands.^60^ Nevertheless, one of the first examples of modulator induced defectivity in MOFs was in the copper-isophthalate based NOTT-101, where 1,1′:4′,1′′-terphenyl-3,3′′,5,5′′-tetracarboxylate (tptc) ligands are connected by [Cu_2_(RCO_2_)_4_] paddlewheels into an **nbo** topology framework [Cu_2_(tptc)]_*n*_. Incorporation of monomeric isophthalates functionalized in their 5-positions results in their replacement of the tptc linkers and concurrent formation of defects, where the functionality at the 5-position projects into the pores of the MOF, which also increase in size (Fig. 9). This strategy was used to enhance gas uptake capacity, through increased porosity, and induce greater interactions with the sorbate, through the pore-projecting functionality.^61^

Defectivity in the UiO-66 isoreticular series was first identified in 2011,^60^ but deliberate induction of defectivity through modulation – in this case incorporation of acetic acid into solvothermal syntheses – was reported in 2013. Increasing the acetic acid concentration during synthesis tunes porosity, with *S*_BET_ increasing from 1000 m^2^ g^−1^ to 1620 m^2^ g^−1^ in a tuneable fashion, while inelastic neutron scattering experiments confirmed the acetate was acting as a charge-compensating defect, replacing the 1,4-bdc linker and capping clusters.^62^ Two different studies reported single crystals of UiO-66 with the identification of missing linker defects possible by single crystal X-ray diffraction. In benzoic acid modulated crystals, as many as 27% of the linkers were missing; defects were not capped by benzoate, but instead by solvents, and notably, the UiO-67 analogue showed little sign of defectivity.^33^ In formic acid modulated crystals of UiO-66, missing linker defects were also capped by solvents, with charge balancing counterions detectable in the pores.^63^ The fact that modulators do not cap defect sites in these two single crystal samples suggests modulator incorporation could be a consequence of kinetic control, and that rapid syntheses will promote modulator-based defectivity; both single crystal syntheses took 48 h, whereas defective microcrystalline powder samples are typically prepared overnight.

**Fig. 10:**
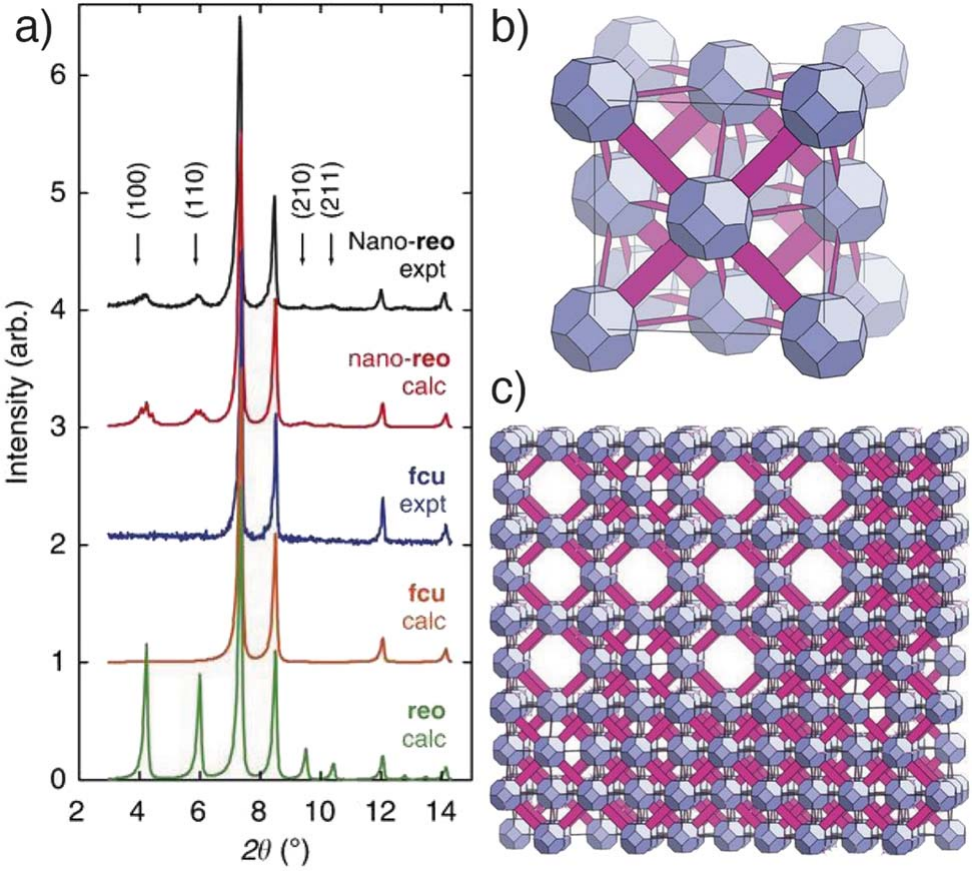
(a) Stacked powder X-ray diffractograms of UiO-66(Hf) samples showing appearance of the **reo** phase in formic acid modulated syntheses (top) compared to calculated patterns for the **fcu**, **reo**, and nano-**reo** (nano domains of **reo** within **fcu**) phases. (b) Image of the non-defective, 12-connected **fcu** net of UiO-66(Hf). (c) Image of missing cluster defects leading to the formation of **reo** nanodomains in UiO-66(Hf). Reprinted (adapted) with permission from ref. 64. Copyright (2014) Springer Nature.

Whilst it may be expected that these defects would be randomly distributed across particles, PXRD analysis (Fig. 10a) of formic acid modulated syntheses of the hafnium congener, UiO-66(Hf) (Fig. 10b), revealed the presence of defect nanodomains (Fig. 10c), where missing cluster defects are correlated to form domains of a new phase, an eight connected defective form of UiO-66 with **reo** topology. The presence of these domains in one particle was unequivocally proven by electron diffraction, and their occurrence increases as the amount of formic acid modulator is increased in syntheses, suggesting formate caps the clusters as a charge-compensating defect ligand. A vacancy fraction of around 13% and domain size of around 5 nm was found for the **reo** defect phase, with coalescence of kinetically formed **reo** domains into larger monoliths of **fcu** phase UiO-66 suggested as a formation mechanism.^64^ Indeed, dual incorporation of benzoate, from benzoic acid modulation, and DMF solvent as defect capping ligands facilitates the phase-pure formation of DUT-51, where eight connected Zr_6_ SBUs link dithieno[3,2-*b*;2′,3′-*d*]-thiophene-2,6-dicarboxylate (dttdc) into the same **reo** net with overall formula [Zr_6_O_6_(OH)_2_(dttdc)_4_(C_6_H_5_COO)_2_(DMF)_6_], although the benzoate and DMF are crystallographically disordered and not well resolved.^65^

**Fig. 11:**
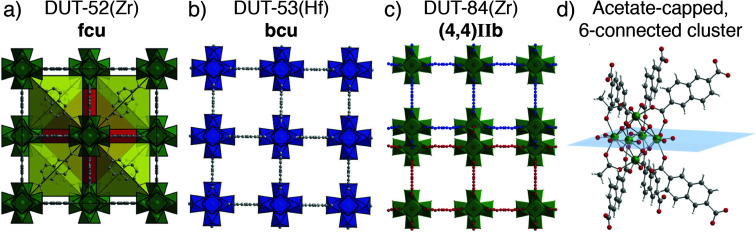
Crystal structure images of the MOFs formed by 2,6-ndc and Zr/Hf under acetic acid modulation. (a) DUT-52 (Zr); 12-connected **fcu** net. (b) DUT-53(Hf); 8-connected **bcu** net. (c) DUT-84(Zr); 6-connected **(4,4)IIb** net. (d) The ordered, acetate capped cluster of DUT-84(Zr). Reproduced (adapted) with permission from ref. 66. Published (2013) by The Royal Society of Chemistry.

Fully occupied, ordered modulator molecules also abound in a number of MOF materials. Carboxylic acid modulation of self-assembly of Zr or Hf with 2,6-naphthalenedicarboxylate (2,6-ndc) can tune between three distinct phases. Addition of 47 equivalents of acetic acid facilitates formation of DUT-52(Zr), an isoreticular analogue of UiO-66 with **fcu** topology (Fig. 11a), while 63 equivalents allows isolation of an eight connected material, DUT-53(Hf), with ordered acetate incorporation and an overall formula [Hf_6_O_6_(OH)_2_(2,6-ndc)_4_(CH_3_COO)_2_(Solvent)_6_]_*n*_. This **bcu** net (Fig. 11b) could also be isolated by benzoic acid modulation, albeit with a change in crystal symmetry from tetragonal to orthorhombic induced by steric repulsion of the 2,6-ndc linker by the larger benzoate ligand. Addition of 254 equivalents of acetic acid leads to DUT-84(Zr), a six-connected structure with an effectively two dimensional **(4,4)IIb** net (Fig. 11c), with overall formula [Zr_6_O_8_(2,6-ndc)_3_(CH_3_COO)_2_(solvent)_8_]_*n*_.^66^ The acetate ligands again cap the clusters (Fig. 11d), showing that modulator incorporation can dramatically change the phase that forms in specific syntheses, for example facilitating formation of new phases where steric hindrance dictates lower connectivity clusters are required.^67^

A number of archetypal and regularly used MOFs now rely on this principle, for example MOF-808, where 1,3,5-btc is linked by Zr_6_ SBUs with significant amounts of incorporated, ordered formate modulator in the final material, [Zr_6_O_4_(OH)_4_(1,3,5-btc)_2_(HCOO)_6_]_*n*_.^68^ The fact that the commonly used formamide solvents react thermally with water to release formate^69^ should, therefore, always be taken into account in assessing defectivity of MOFs synthesized in these media.

### 3.2. Applications of modulation-induced defective MOFs

**Fig. 12:**
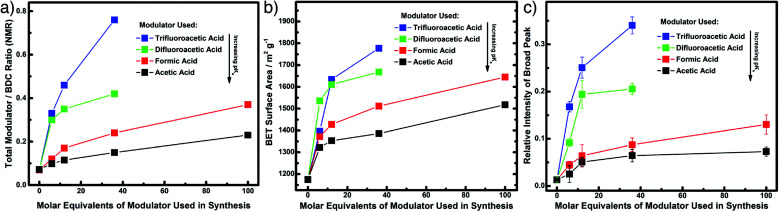
Close alignment of different properties of samples of UiO-66 modulated with differing carboxylic acids. (a) Total modulator incorporation from ^1^H NMR spectra of digested samples. (b) BET surface areas from N_2_ adsorption/desorption isotherms. (c) Presence of the **reo** defect phase from intensity of peaks in powder X-ray diffractograms. Reprinted with permission from ref. 70. Copyright (2016) American Chemical Society.

Whether ordered or not, the ability to tune pore size and chemistry using defectivity has led to the exploration of a wide range of applications of defective MOFs, and this section will detail a representative set of examples covering modulation-induced defectivity. Porosity is a physical property that obviously depends heavily on defectivity; a comprehensive study of the modulation of UiO-66 showed that both modulator incorporation, and therefore porosity, can be tuned by modifying the p*K*_a_ of the modulator carboxylic acid moiety. Examining modulation with acetic (p*K*_a_ = 4.76), formic (p*K*_a_ = 3.77), difluoroacetic (p*K*_a_ = 1.24), and trifluoroacetic (p*K*_a_ = 0.23) acids, it was found that the lower the p*K*_a_ of the modulator, the more defectivity it induced. ^1^H NMR spectra of acid digested samples showed increasing modulator to 1,4-bdc ligand ratios as the p*K*_a_ decreased (Fig. 12a), but with appreciable amounts of formate in each sample as a consequence of solvent decomposition. An analogous increase in porosity was observed through N_2_ uptake (Fig. 12b), with S_BET_ as high as 1777 m^2^ g^−1^ when 36 equivalents of trifluoroacetic acid are added to syntheses, alongside a concomitant increase in intensity of Bragg reflections for the defective **reo** phase in powder X-ray diffractograms (Fig. 12c).^70^

Similarly, use of a larger modulator – dodecanoic acid – results in UiO-66 materials with hierarchical porosity, showing both micro- and mesopores which likely form as a consequence of the modulator being larger than the 1,4-bdc linker.^71^ Dodecanoate-modulated UiO-66 has subsequently been utilized for uptake of uranium from seawater, with enhanced capacities and adsorption kinetics compared to non-defective samples.^72^ Hierarchical porosity by modulator incorporation has also been induced in Cu,^73–75^ Sc,^76^ and Fe^77^ MOFs, while samples of MOF-5 with micro-, meso-, and macropores can be prepared by modulation with 4-dodecylbenzoic acid, although the modulators do not remain within the MOFs after activation.^78^

As well as enhancing porosity, improvements in dye adsorption were obtained with benzoic acid modulated UiO-66. In this case, the benzoate defects were removed by postsynthetic treatment with aqueous HCl, yielding a material with *S*_BET_ = 1890 m^2^ g^−1^ and significantly enhanced uptake of Safranine T dye compared to the as-synthesised material.^79^ Incorporation of modulators as defects and their subsequent removal without destroying the underlying MOF structure highlights their dynamic coordinative nature. For example, it has been shown that formate defects in UiO-67 can be exchanged with amino acids as a pore-functionalisation protocol, or even with bpdc linkers to “repair” the crystal into a non-defective pristine MOF.^80^ Exchange of the ordered formate modulators in MOF-808 for sulfate anions is also possible, by suspension of the MOF in dilute aqueous sulfuric acid solutions, generating materials with the composition [Zr_6_O_5_(OH)_3_(1,3,5-btc)_2_(SO_4_)_2.5_(H_2_O)_2.5_]_*n*_. The sulfates coordinated to the Zr clusters endow the MOF with superacidity – the Hammett acidity function *H*_0_ was found to be less than −14.5 – and the MOF catalyzed both the Friedel–Crafts acylation of anisole and isomerization of α-pinene.^68^

**Fig. 13:**
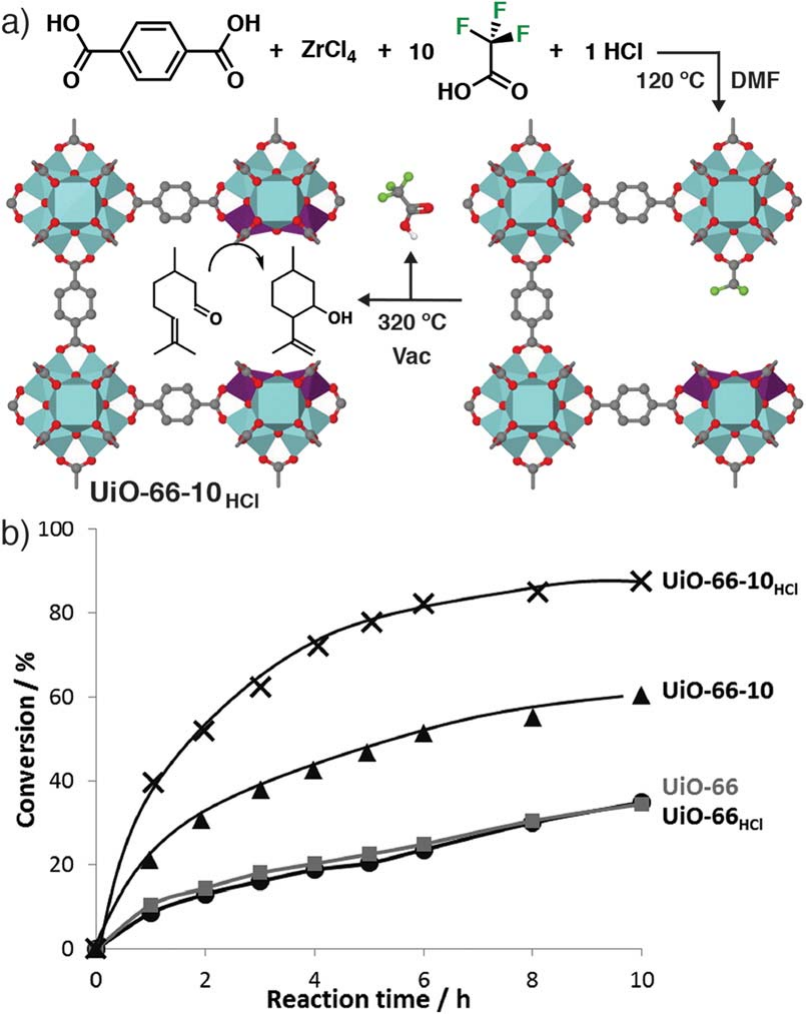
(a) Synthesis of a Lewis Acid catalyst based on defective UiO-66, by postsynthetic thermal removal of incorporated trifluoroacetate modulators. (b) Enhanced catalytic activity of the TFA-modulated MOFs, UiO-66-10_HCl_ and UiO-66-10, compared to unmodulated analogues UiO-66 and UiO-66_HCl_, in the cyclisation of citronellal. Reprinted (adapted) with permission from ref. 81. Copyright (2013) American Chemical Society.

Thermal removal of trifluoroacetate defects from UiO-66 has been used to create a Lewis Acid catalytic material. Co-modulation with 10 equivalents of TFA and one equivalent of HCl, followed by postsynthetic heating to 320 °C for 12 h (Fig. 13a), yielded the most catalytically active material – UiO-66-10_HCl_ – in the cyclisation of citronellal to isopulegol compared to analogues without TFA and/or HCl (Fig. 13b). Solid-state ^19^F NMR spectroscopy confirmed the presence of chemi- and physisorbed TFA in the as-synthesised material, which was completely removed after heating to 200 °C. IR spectroscopy confirmed cluster dehydroxylation at the elevated temperature, while chemisorption of acetonitrile could monitor the number of available Lewis acid sites as a function of activation temperature, saturating at a value which suggested two defect vacancies per Zr_6_ cluster. The catalyst was also active in the Meerwein reduction of 4-*t*-butylcyclohexanone with isopropanol, and conversions of 93% could be achieved with a nitro-substituted analogue, compared to only 7% conversion with a non-defective sample.^81^ A similar concept has also been applied to removal of acetate defects from the Zr MOFs UiO-66, MOF-808, and DUT-84 by microwave heating in aqueous media. The activated samples showed enhanced catalytic hydrolysis of the organophosphate nerve agent VM, presumably as a consequence of the increase in available Lewis Acid sites.^82^

Modulators functionalized with catalytic units can also be incorporated at defect sites. The use of l-proline as a modulator, which had previously been shown to produce high quality, defect-free single crystals of Zr MOFs,^44,45^ could also lead to defective UiO-66 with appreciable quantities of l-proline – a chiral organocatalyst – trapped at defects sites, by modifying synthetic conditions. In the diastereoselective aldol addition of cyclohexanone to 4-nitroacetophenone, l-proline itself resulted in a 61% conversion in the homogenous reaction, in comparison to 100% conversion by the MOF with l-proline anchored at defect sites. The diastereoselectivity of the MOF-based catalyst was also notably enhanced, suggesting not only that heterogeneous MOF catalysts can be prepared by binding functionality at defect sites, but that the local pore environment can influence regio- and stereoselectivity of the products, much like the active sites of enzymes.^83^

Defects can modify other materials properties of MOFs. The mechanical stability of a series of modulated UiO-66 samples containing monocarboxylates as charge compensating defects was assessed by examining porosity and crystallinity after ball milling for set times. Mechanical stability typically has an inverse relationship with porosity, but samples with significant modulator incorporation, which usually increases porosity, were found to also have enhanced mechanical stability. This effect was related to the p*K*_a_ of the modulator being incorporated, becoming more pronounced going from acetic acid (p*K*_a_ = 4.76), to chloroacetic acid (p*K*_a_ = 2.87), to trifluoroacetic acid (p*K*_a_ = 0.23), and was hypothesized to originate in a local electron withdrawing effect of the more acidic modulators increasing the electropositivity of the Zr^4+^ centres and thus strengthening the bonds between the Zr ions and the 1,4-bdc linkers. Evidence for this was found in the IR spectra of the materials, with a band assigned to the Zr–OC asymmetric vibration moving to higher wavenumber as the p*K*_a_ of the defect modulator decreased, suggestive of increasing bond strength.^84^

**Fig. 14:**
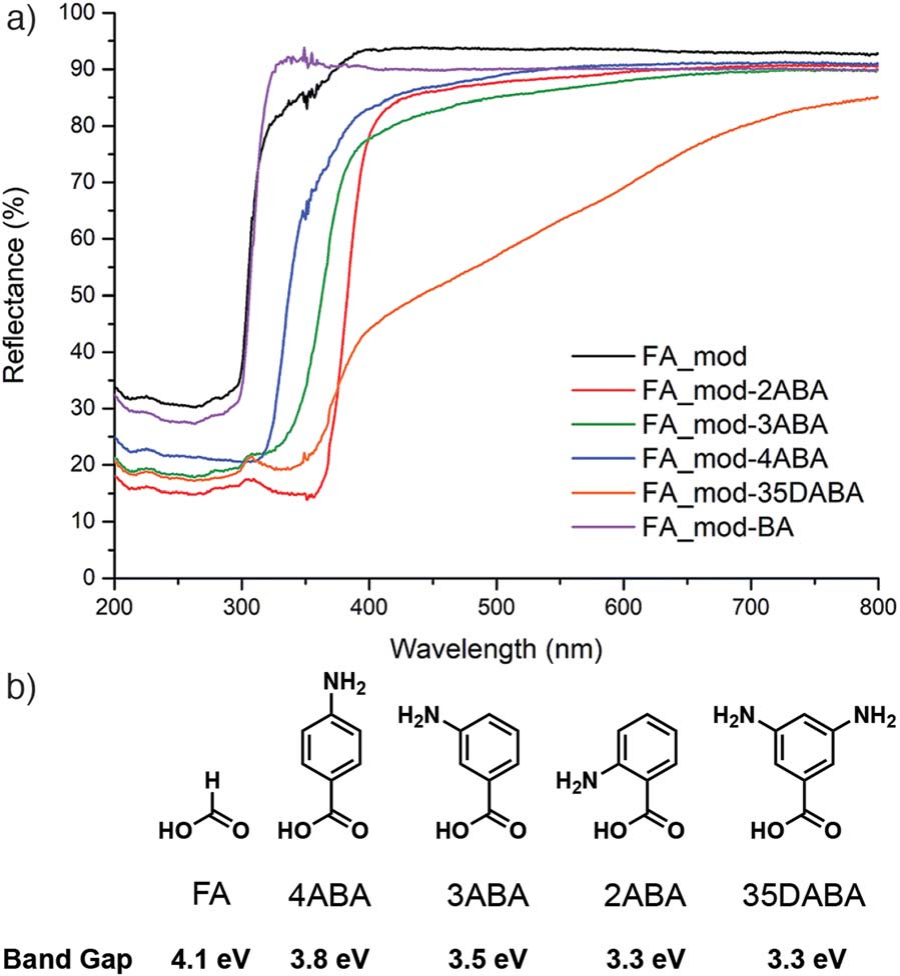
(a) Diffuse reflectance UV/Vis spectra of formic acid modulated UiO-66 samples before (FA_mod) and after exchange of formate defects with (b) different aminobenzoic acids, and the resultant experimental band gaps. Reproduced (adapted) with permission from ref. 85. Published (2019) by The Royal Society of Chemistry.

It is also possible to modify the band gap of Zr MOF samples through defect functionalization. Again taking advantage of the dynamic nature of defects, formate-modulated UiO-66 was functionalized at its defect sites by postsynthetic exchange of modulators with amino-substituted benzoic acids through immersion in DMF solutions. Experimentally measured band gaps red-shifted by up to 0.8 eV as the electron-donating ability of the aminobenzoate increased (Fig. 14), while mechanical stability decreased, mirroring the previous study. The lowest band gap material functionalized with 3,5-diaminobenzoate (*E*_g_ = 3.3 eV) showed enhanced photocatalytic behaviour in both the gaseous reduction of CO_2_ to CO and degradation of Rhodamine B in aqueous solutions compared to the parent, formate-modulated MOF (*E*_g_ = 4.1 eV).^85^

Modulator incorporation and defectivity also enhance a number of properties desirable for the application of MOFs in nanoscale drug delivery. In the modulated synthesis of UiO-66 nanoparticles, Mirkin *et al.* found that the amount of modulator added, and its p*K*_a_, can tune particle size into the nanoregime. Investigating acetic acid (p*K*_a_ = 4.87), formic acid (p*K*_a_ = 3.77), dichloroacetic acid (p*K*_a_ = 1.35), and trifluoroacetic acid (p*K*_a_ = 0.23), it was found that particle size increased with modulator concentration in solvothermal syntheses in DMF, with the more acidic modulators, dichloroacetic and trifluoroacetic acid, requiring lower concentrations to induce this effect, in line with previous studies. The samples modulated with formic, dichloroacetic and trifluoroacetic acid also showed colloidal stability in aqueous solutions by dynamic light scattering, with aggregation of the acetic acid modulated MOFs depending on modulator incorporation. This can be rationalized by measuring zeta potentials, where larger numbers of modulator incorporation defects increase surface charge, and hence interparticle repulsion, to stabilize the colloidal MOFs.^86^

**Fig. 15:**
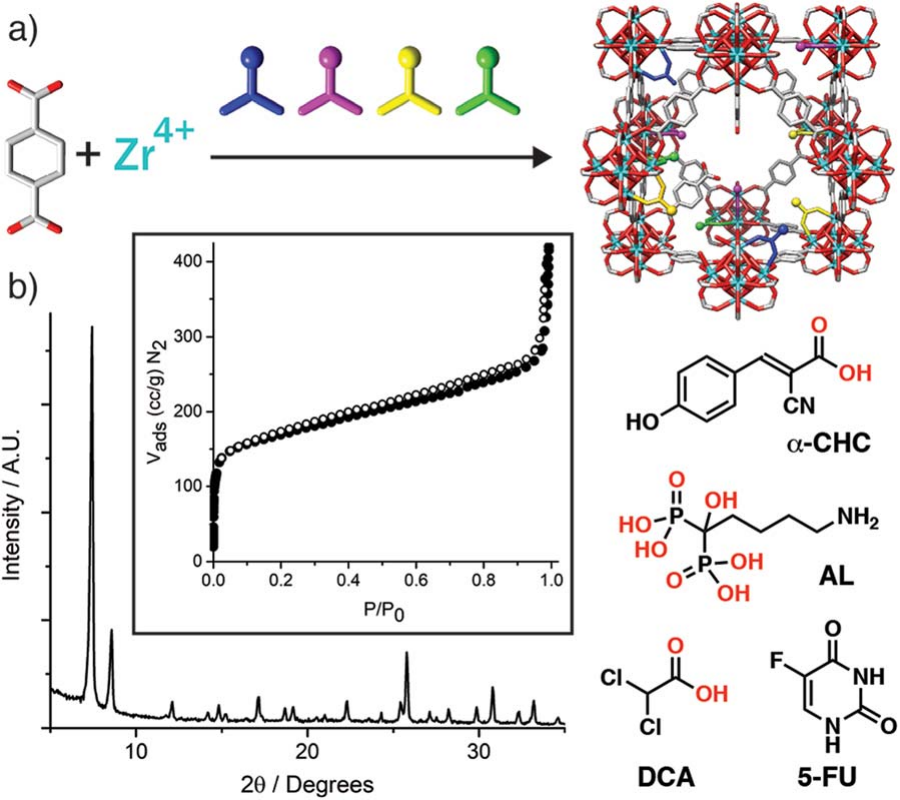
(a) Schematic of the multivariate modulation protocol. (b) Retention of crystallinity and porosity (inset) of UiO-66 when defect-loaded by multivariate modulation with three different drug molecules, α-CHC, AL, and DCA, allowing postsynthetic loading of a fourth, 5-FU. Reprinted (adapted) with permission from ref. 90. Copyright (2020) John Wiley & Sons.

Colloidally stable porous nanoparticles are obviously highly desirable for drug delivery, and our group has taken this a step further by using drug molecules as modulators in the synthesis of UiO-66 and its isoreticular analogue, Zr-fum, to “defect-load” chemotherapeutics into MOFs. Dichloroacetate can induce anticancer cytotoxicity if it is endocytosed and escapes lysosomal degradation; the low p*K*_a_ of dichloroacetic acid means it can be incorporated in significant quantities (up to 20% w/w) by modulation while controlling particle size and dispersion.^87,88^ The MOFs maintain their high porosities, as a consequence of the dichloroacetate being defect-loaded rather than adsorbed into pores, meaning an additional drug such as 5-fluorouracil can be postsynthetically loaded to create dual-drug delivery systems with synergistic anticancer cytotoxicity.^89^ It is even possible to defect-load more than one drug using a technique we have termed “multivariate modulation”, by simply adding up to three distinct modulators into solvothermal syntheses of UiO-66 (Fig. 15a). Dichloroacetic acid, the bis-phosphonate alendronate, and α-cyano-4-hydroxycinnamic acid were all incorporated into UiO-66 as defects, with relative incorporation again related to modulator p*K*_a_ and coordinating units (phosphonate *vs.* carboxylate). Despite 25% (w/w) of the solid being defect-loaded drugs, porosity was maintained to load 5-fluorouracil (Fig. 15b), creating a quaternary drug cocktail with enhanced *in vitro* selectivity in anticancer cytotoxicity.^90^ This protocol has also been utilized to incorporate drugs alongside targeting units, such as folate^87^ and a carboxylate-functionalised triphenylphosphonium unit,^91^ to enhance cytotoxicity. If synthesis conditions are strictly controlled, then these additional features can be primarily confined to MOF particle surfaces.

### 3.3. Surface functionalisation

**Fig. 16:**
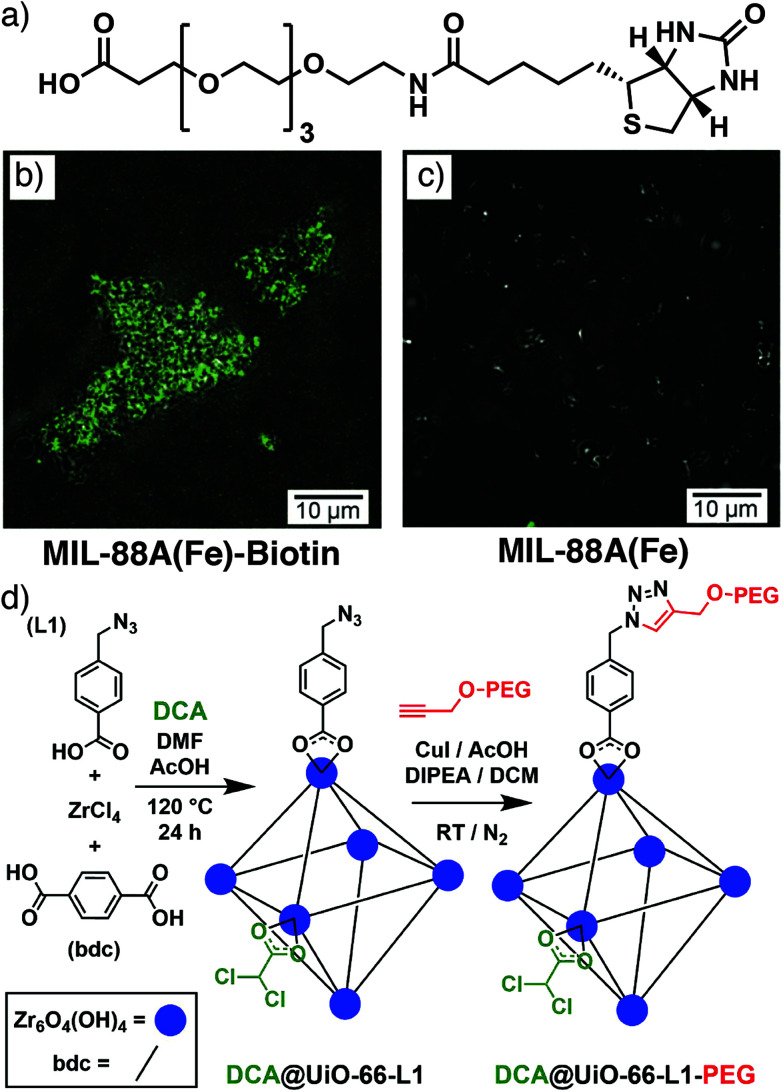
(a) Biotin modulator incorporated onto the surface of MIL-88A(Fe). Overlays of optical and confocal fluorescence microscopy images for (b) MIL-88A(Fe)-Biotin and (c) a MIL-88A(Fe) control after incubation with Alexa-Fluor488-functionalised streptavidin. Reprinted (adapted) with permission from ref. 93. Copyright (2015) John Wiley & Sons. (d) Schematic of the click modulation protocol for stepwise defect-loading of drugs and surface modification of UiO-66. Reprinted (adapted) with permission from ref. 95. Copyright (2017) Elsevier.

Direct observation of the interaction of modulators with growing particle surfaces has previously been described in Section 2, and incorporation of modulators can therefore be confined to MOF particle surfaces, typically if the modulator is significantly larger than the pore windows.^92^ Modulation of the microwave synthesis of MIL-88A(Fe), a fumarate-linked MOF with formula [Fe_3_O(fum)_3_(OH_2_)_2_X]_*n*_ where X is a monoanion, typically OH^−^, Cl^−^, or F^−^, with larger modulators resulted in their localization on particle surfaces. 4-Trifluoromethylcyclohexanoic acid, a carboxylic acid-functionalized poly(ethylene glycol) (PEG) with molecular weight around 2000 Da, and a carboxylic acid-functionalised biotin with a tetra(ethylene glycol) spacer (Fig. 16a) were all utilized, with the fluorine atoms of 4-trifluoromethylcyclohexanoic acid allowing monitoring of surface functionalization by X-ray photoelectron spectroscopy. The availability of the biotin functionality on particle surfaces was demonstrated by aggregation of the particles when exposed to streptavidin, which can bind up to four biotin molecules, and also by surface fluorescence when streptavidin tagged with the green dye Alexa-Fluor488 was introduced (Fig. 16b). These effects were not observed in particles without biotin (Fig. 16c).^93^ Particle size decreased as larger amounts of the PEG chain were included in synthesis, and this size control through capping was subsequently investigated for PEGs of different molecular weights. Particle size decreased from ∼1000 nm in length to ∼300 nm as the PEG molecular weight increased across the series 178, 588, ∼2000, and ∼5000 Da, but use of a larger PEG with molecular weight ∼20 000 Da did not further decrease the size. The polymer coatings allowed controlled release of sulforhodamine B compared to uncoated materials.^94^

We have introduced a modulator-based surface functionalization protocol – click modulation – whereby functionalized modulators are incorporated into MOFs, primarily at particle surfaces, and postsynthetically modified by so-called “click” reactions. Both *p*-azidomethylbenzoic acid (azide) and *p*-propargyloxybenzoic acid (alkyne) were incorporated into UiO-66 and their functionality was available for further transformation by copper-catalysed azide–alkyne cycloaddition (CuAAC) with both small molecules and large polymers, indicating the modulators were predominantly on the surfaces of the MOF nanoparticles. This two-step protocol (Fig. 16d) is also compatible with defect drug loading, by co-modulating with, for example, dichloroacetic acid, and the addition of surface PEG chains enhances stability, induces pH responsive cargo release, and dramatically enhances the *in vitro* anticancer cytotoxicity of the dichloracetate cargo by altering cancer cell endocytosis mechanisms.^95^

Similar results have been demonstrated by modulation of MIL-88A(Fe) with 10-undecynoic acid, followed by CuAAC with azide-functionalised coumarins to allow detection of surface functionalization by fluorescence microscopy.^96^ Enhanced incorporation of UiO-66-NH_2_ particles into mixed matrix membranes has also been achieved through modulation with 4-aminobenzoic acid; the modulators were located at least in part on the particle surfaces, providing an additional functional handle, alongside that of 2-NH_2_-1,4-bdc, for covalent crosslinking with Matrimid polymer to produce membranes with improved stability.^97^

This diversity of the chemistry of modulator-induced defects, located either through the bulk of the MOF or confined to particle surfaces, shows the potential for enhancing materials properties and enabling new applications. Defects are clearly dynamic, and therefore sites for carrying out further reactivity or exchange. The majority of current research is related to Zr MOFs, in particular UiO-66, which leaves notable opportunities to investigate the defect chemistry of other important families of MOFs while at the same time developing their modulated self-assembly.

## 4. Phase selection

**Fig. 17:**
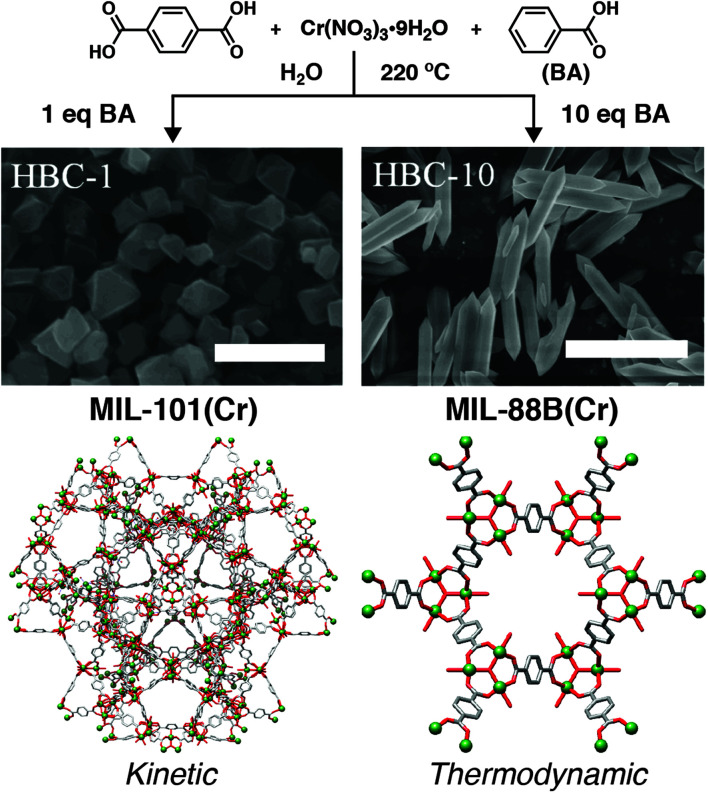
Schematic of benzoic acid (BA) modulated hydrothermal syntheses of Cr(iii) MOFs linked by 1,4-bdc, showing selection of the thermodynamic MIL-88B(Cr) phase (right, hexagonal rods, scale bar 3 μm) over the kinetic MIL-101(Cr) phase (left, octahedra, scale bar 500 nm) as the modulator content in syntheses increases. Reproduced (adapted) with permission from ref. 98. Published (2019) by The Royal Society of Chemistry.

There are large numbers of metal–ligand combinations that can result in multiple framework structures – for example, there are at least 5 possible Fe(iii) 1,4-bdc MOFs that have been prepared by direct synthesis – with isolation of phase pure MOFs often challenging and requiring careful control over reaction conditions. Coordination modulation can potentially influence phase selection from complex mixtures in a number of ways, for example, pre-organising particular discrete metal clusters or by controlling the kinetics of self-assembly through competition for both metal ions and protons. Examining the Cr(iii) 1,4-bdc system, Liu *et al.* found that addition of benzoic acid modulator to hydrothermal syntheses could influence the phase that was isolated (Fig. 17). Unmodulated syntheses resulted in the formation of MIL-101(Cr), which is expected to be the kinetic product of this metal–ligand system, and addition of up to 3 equivalents of benzoic acid to syntheses gradually reduced particle size while enhancing porosity. When 5 equivalents of benzoic acid is added, MIL-88B(Cr), which has the same metal cluster and overall composition of MIL-101(Cr) but a more dense structure, appears in a mixture of the two phases, while 10 equivalents yields solely MIL-88B(Cr).^98^ These results indicate that modulation can influence the kinetics of self-assembly. Both phases have identical composition and SBU, but differ in topology; MIL-88B(Cr) is more dense, and so thought to be the thermodynamic product. This suggests that coordination modulation can select for the thermodynamic product by competing with ligands for metal ion and proton, effectively slowing the self-assembly process.

Our group has observed a similar phase selection in a related system: Fe(iii) MOFs linked by bpdc. Unmodulated solvothermal syntheses in DMF resulted primarily in the MIL-88D(Fe) phase, a non-interpenetrated isoreticular analogue of MIL-88B with formula [Fe_3_O(bpdc)_3_(OH_2_)_2_X]_*n*_ where X is a monoanion. When various carboxylate modulators are added to syntheses, the two-fold interpenetrated polymorph MIL-126(Fe) is the major product, with both acetic and formic acid proving particularly effective at generating this denser phase. DFT calculations confirmed MIL-126(Fe) is the thermodynamic product and MIL-88D(Fe) the kinetic one (increased reaction temperature also favours the latter) again showing that coordination modulation has modified reaction kinetics to select for the thermodynamic product.^77^

Aluminium MOFs linked by 1,3,5-btc have also been shown susceptible to modulation. In the absence of modulator, microwave heating of aqueous solutions of aluminium nitrate and 1,3,5-btc to 210 °C resulted in mixtures of MIL-100(Al) and MIL-96(Al). Lower reaction pH is thought to favour the discrete trimeric clusters found in MIL-100, [Al_3_O(1,3,5-btc)_2_(OH_2_)_2_X]_*n*_ (X = a monocounterion), but addition of three equivalents of acetic acid surprisingly generated phase pure MIL-96(Al), which has infinite one-dimensional chain SBUs and overall formula [Al_12_O(OH)_18_(H_2_O)_3_(Al_2_(OH)_4_)(1,3,5-btc)_6_]_*n*_. In this case, it would be expected that MIL-96(Al) would be the thermodynamic product, being considerably more dense, and so it is likely that acetic acid induces kinetic control through coordinative competition (*i.e.*, coordination modulation) rather than modifying pH (*i.e.*, pH modulation) to dictate the final product.^99^

Barea *et al.* discovered a new phase when attempting to control the particle size of CYCU-3, an aluminium MOF linked by stilbene-4,4′-dicarboxylate (sdc) into a MIL-68 topology material with composition [AlOH(sdc)]_*n*_, by modulation with acetic acid. Incorporation of 50 equivalents of acetic acid to solvothermal syntheses in DMF resulted in the isolation of a new, highly defective material whose structure was solved by continuous rotation electron diffraction. The new phase consists of corner sharing AlO_6_ octahedra connected by sdc ligands into a topology observed in the archetypal [Sc_2_(1,4-bdc)_3_]_*n*_ MOF, but with significant defectivity attributed to incorporation of acetate as a charge-compensating defect. This new phase was found to be denser than the targeted CYCU-3, suggesting again that modulation has induced the formation of the thermodynamic product, albeit with significant defectivity, and that CYCU-3 is the kinetic product.^100^

**Fig. 18:**
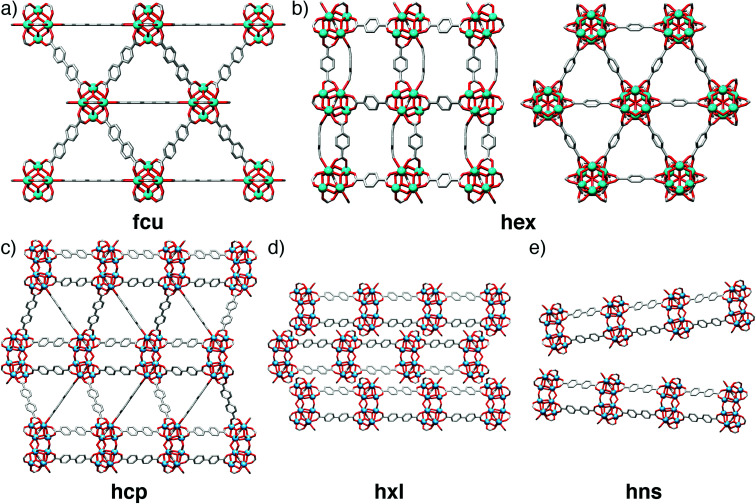
Solid-state structures of different topologies accessible within the phase space of Zr MOFs linked by linear dicarboxylate ligands. (a) UiO-67 in the **fcu** topology. (b) The Zr-bdc MOF which is a polymorph of UiO-66 but has **hex** topology, viewed down the *b* (left) and *c* (right) crystallographic axes. (c) The **hcp** phase formed by Hf and bpdc. (d) The **hxl** phase formed by Hf and bpdc, which can spontaneously arise from the **hcp** phase by loss of bpdc. (e) The **hns** phase of Hf and bpdc, which is a delaminated version of the **hxl** phase.

The phase space for tetravalent Zr/Hf materials linked by linear dicarboxylates has been significantly expanded by modulation with carboxylic acids, and a number of new structures identified. As well as the twelve-connected face-centred cubic (**fcu**) UiO-66 topology MOFs (Fig. 18a), and their defective 8-connected **reo** analogues described previously,^64^ it has been possible to isolate an eight-connected polymorph of UiO-66 with a **hex** net (Fig. 18b),^101^ hexagonal close packed (**hcp**) phases connected by condensed [M_12_O_8_(μ_3_-OH)_8_(μ_2_-OH)_6_(RCO_2_)_18_] clusters (Fig. 18c)^102–104^ which can spontaneously transform into layered **hxl** phases (Fig. 18d), and finally hexagonal nanosheets (**hns**) phases (Fig. 18e) that represent delaminated analogues of the **hxl** phases.^102,103^

The **hcp** phase of UiO-67(Hf), [Hf_12_O_8_(OH)_14_(bpdc)_9_]_*n*_ (Fig. 18c) was synthesized by formic acid modulation of solvothermal syntheses in DMF at high temperatures – below 120 °C the synthesis yields primarily unreacted bpdc linker – and optimum results were obtained at 150 °C using 1 ml of formic acid and 0.05 ml water per 4 ml of DMF. UiO-67(Hf) **hcp** spontaneously transformed into the layered **hxl** phase (Fig. 18d), through loss of three bpdc ligands per Hf_12_ SBU and their replacement with formate; grinding or sonication of either the **hcp** or **hxl** phases led to delamination to form the **hns** material (Fig. 18e).^102^ The importance of water content is again underlined by the subsequent direct preparation of the UiO-67(Hf) **hns** phase by simply quadrupling the water content in the initial synthesis.^103^ The analogous **hcp** phase of UiO-66(Hf) was subsequently prepared by similar high temperature, formic acid modulated syntheses but with addition of significant amounts of water; phase pure materials were isolated with 4 ml DMF, 1.5 ml formic acid and 0.4 ml water. An isostructural **hcp** phase linked by 2,3,5,6-tetrafluoro-1,4-benzenedicarboxylate could be synthesized for both the Zr and Hf congeners in aqueous media with differing quantities of acetic or formic acid modulators, also at high temperatures.^103^ In contrast, the **hcp** phase of UiO-68 (an extended analogue with the tpdc linker) was isolated by using acetic acid as modulator (0.75 ml in 10 ml DMF) at the lower temperature of 90 °C.^104^ The **hex** polymorph of UiO-66 (Fig. 18b) was isolated using 3.6 equivalents of methacrylic acid as modulator, but also Zr(PrO)_4_ as metal source in 1-propanol, indicating that new phases may be discovered by modifying metal source as well as modulator, an approach that has recently led to breakthroughs in the discovery of Ti^4+^ MOFs.^105^

The versatility of the modulation approach is evident in our own study of yttrium MOFs linked by 2,6-ndc, where the addition of different modulators results in six distinct structures for one metal–ligand combination. In some cases the modulators were incorporated into the structures (*e.g.* acetate) but in others they have clearly selected a particular phase, perhaps through templation.^106^ These emerging examples make it clear that coordination modulation is potentially a very powerful structure-directing protocol, allowing isolation of novel materials or selection of phase pure MOFs from complex mixtures. Given its more common use in modifying physical properties or enhancing crystallinity, it is clear that the influence of modulator on the kinetics of self-assembly must also be considered during MOF synthesis.

## 5. Summary and future perspectives

The fine control that coordination modulation offers the synthetic chemist means it is now commonplace in MOF chemistry. From initial attempts to control particle size of MOFs, the technique has grown into a collection of wide-ranging synthetic protocols to facilitate synthesis, produce single crystals, control multiple physical properties, induce defects, and more recently to offer phase control in complex systems. Related techniques are now also being developed, for example click modulation to concurrently control particle size and surface chemistry, and multivariate modulation to enhance pore complexity and store multiple cargo molecules within the porosity of MOFs. The ability to control self-assembly kinetics is potentially very powerful, and so coordination modulation is now inspiring alternative approaches to exert kinetic control, whilst also being applied to alternative materials. For example, coordination modulation has been combined with the preformation of SBUs to access a wide range of Fe(iii) and mixed-metal MOFs as single crystals, in a process that has been termed kinetically tuned dimensional augmentation. By using [Fe_2_M(μ_3_-O)(CH_3_COO)_6_], where M = divalent Fe, Mn, Co, Ni or Zn as a metal source, along with acetic acid as modulator in solvothermal syntheses, 34 MOFs were obtained as large single crystals, allowing a detailed examination of their solid-state structures.^67^

**Fig. 19:**
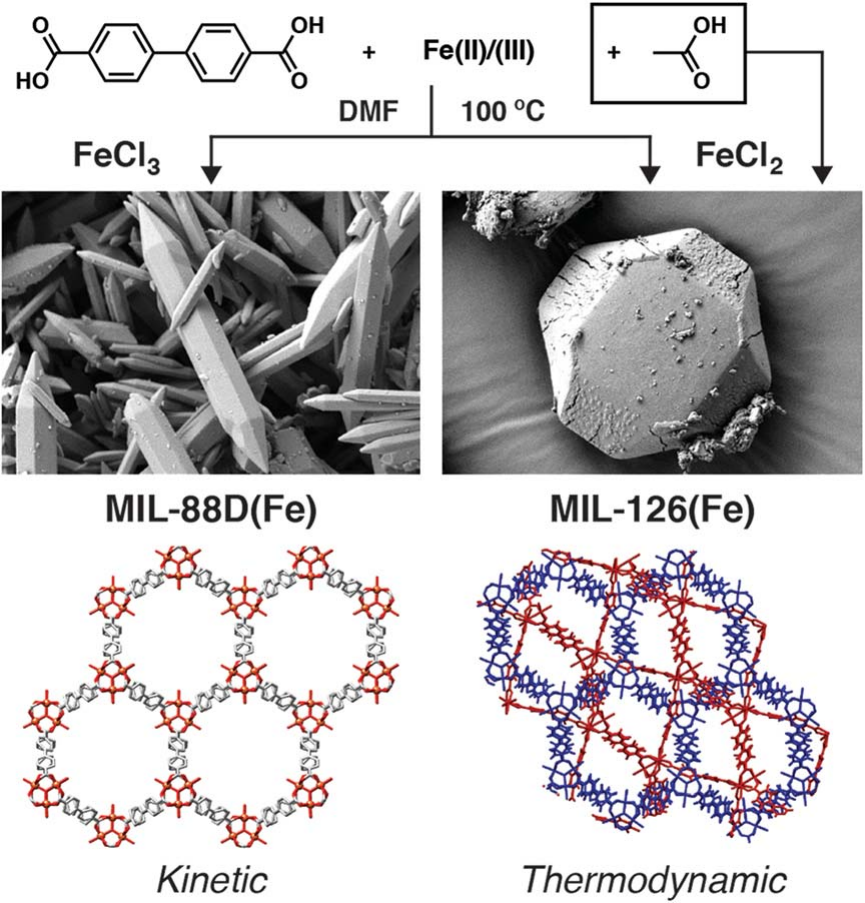
Schematic of the oxidation modulation protocol to select between kinetic and thermodynamic phases in the self-assembly of Fe-bpdc MOFs, which mirrors the control offered by coordination modulation. Reprinted (adapted) with permission from ref. 77. Copyright (2019) American Chemical Society.

Additional kinetic barriers can be imposed by using metal precursors in different oxidation states to those found in the desired MOF. This process, which we have termed “oxidation modulation”,^77^ has been applied to the synthesis of Mn MOFs possessing different structures^107^ and also to tuning between polymorphs in the previously discussed MIL-88D(Fe)/MIL-126(Fe) system. Using Fe^2+^ as a starting material for the Fe^3+^ MOFs introduces an additional necessary autoxidation step that selects for the thermodynamic product, and produces MIL-126(Fe) when FeCl_2_ is used as the starting material, compared to the kinetic product MIL-88D(Fe) when FeCl_3_ is used under identical conditions (Fig. 19). These results replicate the kinetic control offered by coordination modulation; combination of both techniques results in very high quality MIL-126(Fe) with optimal porosity.^77^

Even ditopic modulators are now being introduced; oxalate, which is the smallest possible dicarboxylate and can easily form coordination polymers as a linker, coordinates in a bidentate fashion to single metal ions through one O-donor from each carboxylate, blocking coordination sites. It cannot however, bridge two metals that are also bridged by a μ_2_-OH ligand, as found in many trivalent MOF structures, and so oxalic acid is an excellent modulator of Al MOFs such as MIL-53(Al), formula [AlOH(1,4-bdc)]_*n*_, significantly enhancing crystallinity and particle size.^108^

**Fig. 20:**
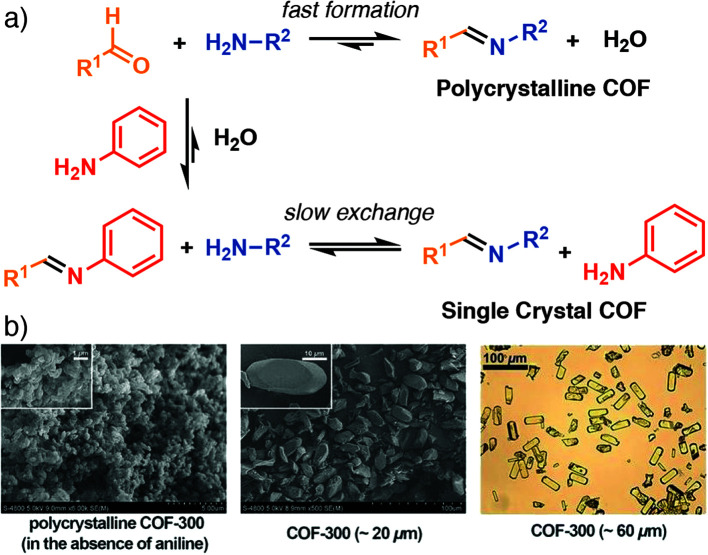
(a) Schematic of aniline-modulated self-assembly of imine COFs to modify kinetics. (b) Selected SEM and optical images of COF-300 samples illustrating the particle size control offered by modulation. Reprinted in part (adapted) with permission from ref. 110. Copyright (2018) The American Association for the Advancement of Science.

Modulated crystallization has also been applied to covalent organic frameworks (COFs), porous organic materials connected by dynamic covalent organic bonds, where the approach is similar; incorporation of modulators mimicking the chemistry of one of the organic components of the COF into the synthesis. Bein *et al.* have shown that COF-5, where benzene-1,4-diboronic acid is condensed with 2,3,6,7,10,11-hexahydroxytriphenylene to form a material linked by boronate esters, can be modulated by incorporation of additional monoboronic acids. Dramatic enhancement in particle size can be achieved – mirroring the breakthroughs seen in coordination modulation of Zr MOFs – while defectivity can be induced by varying the mount of modulator added. Additionally, incorporation of the monoboronic acid, particularly terminating the outer surfaces of COF crystallites, was also observed.^109^ In a similar manner, Yaghi *et al.* demonstrated that imine-linked COFs, prepared by condensation of aromatic amines and aldehydes, can be modulated by addition of aniline to synthetic mixtures (Fig. 20a). Particle size enhancement was significant enough to allow structure determination by single-crystal X-ray diffraction of a number of COFs for the first time (Fig. 20b), yielding key insights into the atomic level structure and disorder found in such materials.^110^

While the versatility of modulation is evident in it now being applied to the self-assembly of materials beyond MOFs, there are still numerous opportunities for developing coordination modulation, and associated defect chemistry, particularly for MOFs linked by trivalent metals where it is currently understudied. The kinetic control offered by the varying modulation techniques should also facilitate discovery of new materials, even in previously well studied chemical spaces. More mechanistic studies would be welcomed, particularly *in situ* analysis of the various solution processes that occur before and during nucleation. With further understanding of the various driving forces underpinning modulated self-assembly will come greater control over MOF synthesis – a crystallization process that is still inherently stochastic – and therefore significant breakthroughs in the discovery, optimization, and application of these materials.

## Conflicts of interest

There are no conflicts to declare.

## Supplementary Material

SC-011-D0SC01356K-s001
