# Supplementary material for: Modulated self-assembly of metal–organic frameworks
Source: Chem Sci. 2020 Apr 6;11(18):4546–62. doi: 10.1039/d0sc01356k (PMC8159241; doi:10.1039/d0sc01356k)
Supplement: SC-011-D0SC01356K-s001 [file SC-011-D0SC01356K-s001.pdf]

Electronic Supplementary Material (ESI) for Chemical Science.
This journal is © The Royal Society of Chemistry 2020

Modulated Self-Assembly of Metal-Organic Frameworks

Ross S. Forgan*

WestCHEM School of Chemistry, University of Glasgow, Glasgow, UK.
Email: ross.forgan@glasgow.ac.uk

SUPPORTING INFORMATION

Table S1. Names, abbrevations, and chemical structures of MOF and COF ligands discussed in this Perspective. **Page S2**.

Table S2. Names, chemical structures, and pK_a values for the modulators discussed in this perspective. **Page S4**.

Table S1. Names, abbrevations, and chemical structures of MOF and COF ligands discussed in this Perspective.

Abbreviation	Name	Structure
1,4-bdc	1,4-Benzenedicarboxylate	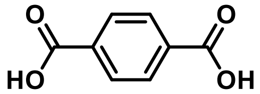
1,4-ndc	1,4-Naphthalene dicarboxylate	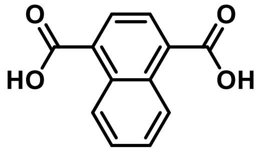
dabco	1,4-Diazabicyclo [2.2.2]octane	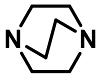
1,3,5-btc	1,3,5-Benzenetricarboxylate	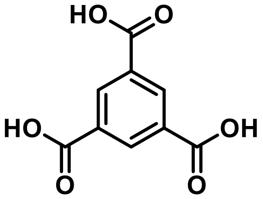
2-NH_2-1,4-bdc	2-Amino-1,4-benzenedicarboxylate	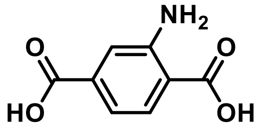
bpdc	Biphenyl-4,4′-dicarboxylate	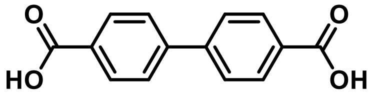
tpdc-NH_2	2′-Amino-1,1′:4,1″-terphenyl-4,4″-dicarboxylate	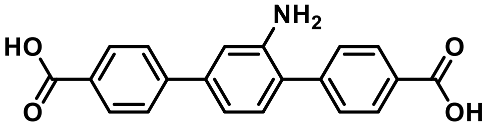
bIm	Benzimidazole	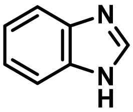
MeIm	2-Methylimidazole	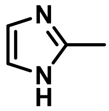
abdc	Azobenzene-4,4′-dicarboxylate	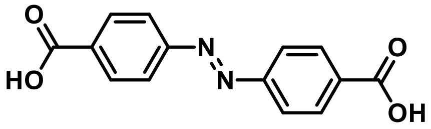
edb	4,4′-Ethynylenedibenzoate	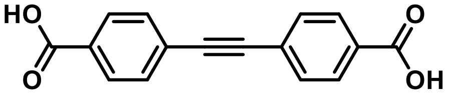
4,4′-bipy	4,4′-Bipyridine	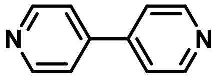

tptc	1,1′:4′,1″-Terphenyl-3,3″,5,5″-tetracarboxylate	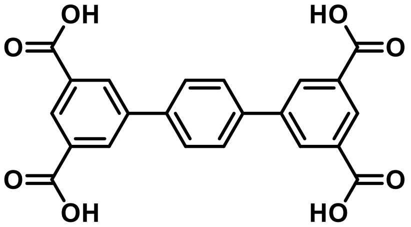
dttdc	Dithieno[3,2-*b*;2′,3′-*d*]-thiophene-2,6-dicarboxylate	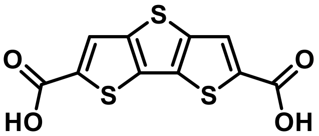
2,6-ndc	2,6-Naphthalene dicarboxylate	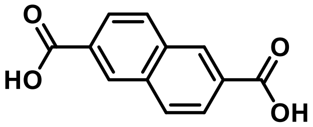
tpdc	1,1′:4,1″-Terphenyl-4,4″-dicarboxylate	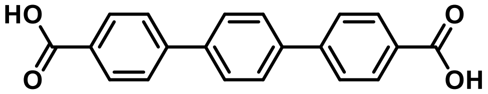
sdc	Stilbene-4,4′-dicarboxylate	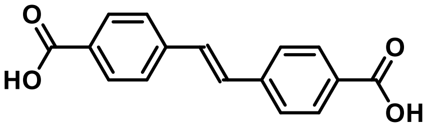
–	Benzene-1,4-diboronic acid	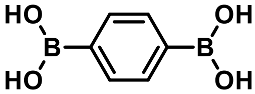
–	2,3,6,7,10,11-hexahydroxytriphenylene	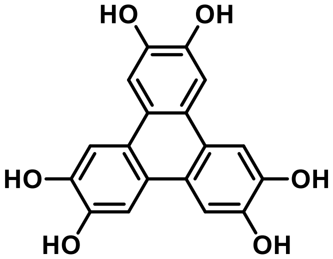

Table S2. Names, chemical structures, and pK_a values for the modulators discussed in this perspective.

**Modulator**	**Structure**	**pK_a (if relevant)**
p-Perfluoromethyl benzoic acid	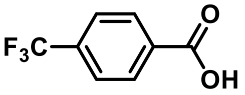	3.69
Acetic acid	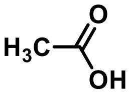	4.76
Pyridine	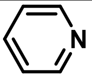	–
Dodecanoic acid	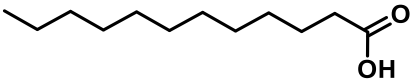	5.30
Benzoic acid	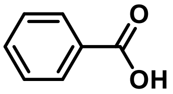	4.20
Perfluorobenzoic acid	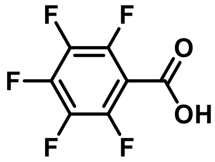	1.60
Stearic acid	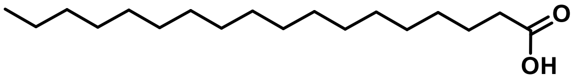	10.15
Sodium formate	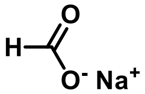	–
Sodium acetate	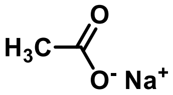	–
Trimethylamine	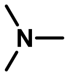	–
Diethylamine	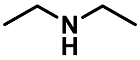	–
1-Methylimidazole	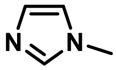	–
n-Butylamine	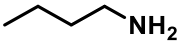	–
4-Decylbenzoic acid	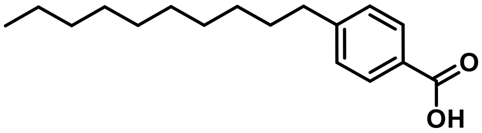	4.35
Formic acid	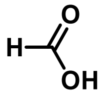	3.77
HCl	HCl	-6.3
L-proline	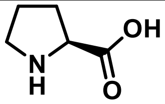	1.99

Difluoroacetic acid	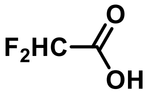	1.24
Trifluoroacetic acid	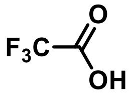	0.23
4-Dodecylbenzoic acid	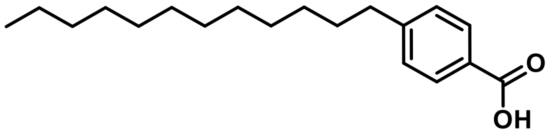	4.48
Chloroacetic acid	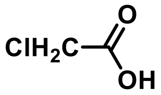	2.87
Dichloroacetic acid	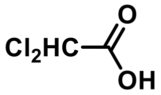	1.35
Alendronate	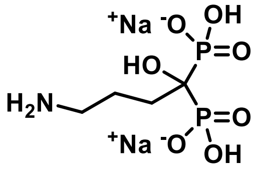	–
α-Cyano-4-hydroxy cinnamic acid	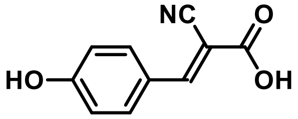	4.90
4-Trifluoromethyl cyclohexanoic acid	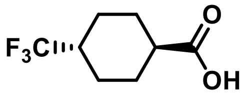	–
PEG-COOH	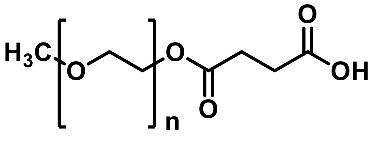 n = 1, 11, 45, 114, 450	–
Biotin-COOH	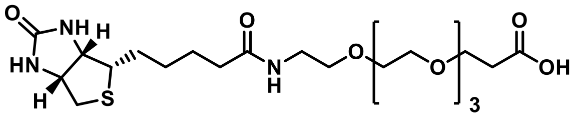	–
4-Azidomethy benzoic acid	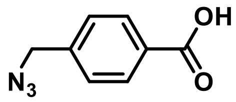	–
4-Propargyloxy benzoic acid	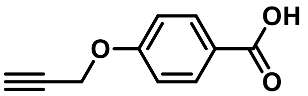	–
Oxalic acid	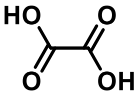	1.46, 4.40
